# The mammalian regulatory architecture: biobehavioral portals and the organization of physiological state

**DOI:** 10.3389/fnins.2026.1923279

**Published:** 2026-10-22

**Authors:** Stephen W. Porges

**Affiliations:** 1 Kinsey Institute, Indiana University, Bloomington, IN, United States; 2 Department of Psychiatry, University of North Carolina at Chapel Hill, Chapel Hill, NC, United States; 3 Department of Psychiatry, College of Medicine-Jacksonville, University of Florida, Jacksonville, FL, United States

**Keywords:** autonomic nervous system, mammalian synapomorphy, neuroception, neuromodulation, physiological state, polyvagal theory, regulatory architecture, vagus nerve stimulation

## Abstract

**Introduction:**

Neuromodulatory interventions can produce overlapping adaptive outcomes despite engaging substantially different biological pathways. Pathway-centered approaches identify how interventions initially engage the nervous system and characterize pathway-specific mechanisms. Convergence across interventions raises an additional question: how can different biological routes influence overlapping domains of adaptive function?

**Methods:**

This conceptual paper develops the Mammalian Regulatory Architecture (MRA), an organizational framework informed by identified mammalian synapomorphies (shared derived characteristics) and comparative anatomical, developmental, physiological, and functional observations.

**Results:**

The MRA hypothesizes that mammalian evolution reorganized relationships among conserved regulatory processes through their integration with mammalian specializations and regulatory demands associated with nursing, prolonged developmental dependency, caregiving, co-regulation, and reciprocal social communication. The framework provides the basis for a biological portal model of neuromodulation that distinguishes the pathway of initial engagement from the functional significance of that engagement within the broader regulatory architecture. Whereas the pathway identifies how an intervention initially engages the nervous system, its portal significance concerns how that engagement influences state-regulating processes within the MRA.

**Discussion:**

The proposed framework reframes neuromodulation from a process defined primarily by its neural target or pathway to one in which pathway engagement may influence broader regulatory organization, physiological state, Accessibility—the state-dependent availability of existing adaptive capacities—and adaptive function. It offers an organizational account of how distinct biological routes may influence overlapping domains of adaptive function.

## 1 Introduction

Neuromodulation has emerged as an increasingly important strategy for influencing health, behavior, emotional regulation, cognitive function, and physiological resilience across a wide range of clinical and non-clinical conditions (Frasch and Porges, 2023; Staats et al., 2024). Contemporary approaches include interventions designed to influence neural activity through identifiable biological pathways, such as vagus nerve stimulation (VNS) and transcutaneous cervical vagus nerve stimulation (tcVNS). Although these interventions enter through identifiable pathways, their reported outcomes frequently extend across multiple physiological and functional domains.

A similar multidomain pattern is observed following interventions that engage the nervous system through substantially different biological pathways. This convergence does not establish a common mechanism. It raises a broader question: How can interventions engaging markedly different biological pathways influence overlapping domains of adaptive function?

This question becomes more consequential when considered in relation to another recurring biological observation. Organisms do not respond to challenge by independently adjusting isolated physiological and behavioral functions. Challenge recruits coordinated changes across regulatory systems. Such coordination is not inherently pathological; it provides an adaptive organization through which resources are mobilized, conserved, or restored in relation to changing demands. Thus, the scientifically relevant observation is not simply that challenge changes many variables, but that these changes occur as an organized response. Different conditions may recruit different configurations of regulation, and their expression may vary with context, history, and physiological condition, while recurring relationships among regulatory functions suggest that this variability occurs within an organized biological system.

The organization of these relationships becomes particularly visible when responses to challenge persist. Across markedly different chronic conditions, recurring burdens may extend across physiological regulation and adaptive function even when the initiating conditions and pathophysiological mechanisms differ substantially. This recurrence raises a question that is difficult to address when each disorder and each manifestation is investigated independently: Why do different forms of challenge repeatedly alter overlapping aspects of physiological and adaptive function?

This problem is particularly visible in conditions described as functional disorders, in which substantial chronic symptoms may persist without identifiable end-organ pathophysiology sufficient to account for their presence, severity, persistence, or distribution. Such conditions challenge diagnostic approaches that rely primarily on demonstrable pathology within the organ in which symptoms are expressed. Importantly, the absence of sufficient end-organ pathology does not imply that symptoms are psychogenic or lack a biological basis. Dysfunction expressed through an organ may also reflect altered neural regulation of end-organ function, whether or not local pathology is present. Functional disorders therefore make particularly visible the distinction between pathophysiology of the end organ and dysfunction in the neural regulation of end-organ function. The location of a symptom does not necessarily identify the location or organizational level of the underlying dysfunction. When persistent symptoms extend across multiple physiological domains, at least some manifestations may reflect alterations in regulatory processes whose influence extends across organs and physiological functions.

From the perspective of discovery science, these observations invite exploration rather than immediate assignment to established disciplinary explanations. A disease-centered approach appropriately asks which mechanisms account for the manifestations of a particular disorder. An exploratory approach asks a complementary question: whether recurring relationships become visible when observations conventionally separated by diagnostic, disciplinary, or mechanistic boundaries are considered together. The recurring organization of these observations provides the empirical problem from which the present framework was developed.

Figure 1 isolates this empirical problem. The recurring pattern does not establish a common regulatory system, common pathology, or common mechanism. Its significance is observational: different challenges with different proximal mechanisms may nevertheless be associated with overlapping patterns of functional burden. Rather than assuming that each manifestation requires an independent explanation, the pattern raises the question of whether some consequences reflect changes in regulatory relationships whose influence extends across functions conventionally investigated separately.

**Figure 1:**
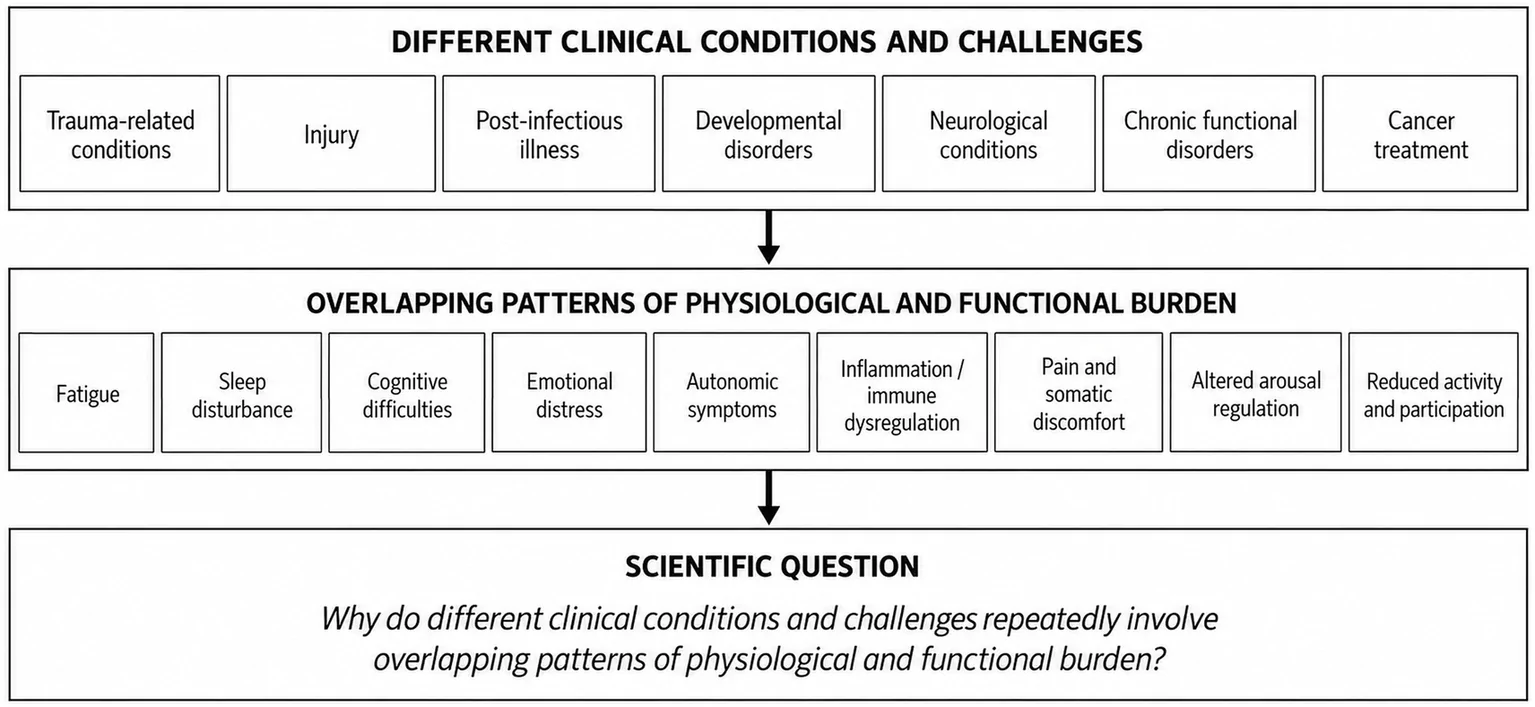
Recurring patterns of functional burden across different challenges. This conceptual illustration depicts overlapping patterns of physiological and functional burden that may occur across different initiating conditions; it does not establish a common mechanism.

Importantly, the recurring observation precedes the explanatory framework and does not depend upon that framework for its identification. The broader question is therefore: What form of regulatory organization could contribute to such recurring patterns without requiring different initiating conditions to share a common pathology or mechanism? The Mammalian Regulatory Architecture (MRA) emerged as a hypothesis developed to address this question.

### 1.1 Discovery, pathway specificity, and hypothesis testing

The empirical pattern illustrated in Figure 1 also identifies a broader methodological issue. Within a particular disorder or biological system, identifying the pathways responsible for specific effects increases mechanistic precision. Anatomical and physiological specification constrains explanation, permits targeted manipulation, and enables competing mechanisms to be distinguished experimentally. Directly identifiable pathways therefore provide an essential foundation for hypothesis testing.

The same specificity can become constraining when an established pathway or mechanism becomes an implicit prerequisite for treating an observation as scientifically informative. Scientific disciplines necessarily organize inquiry around existing knowledge, methods, and explanatory frameworks. These constraints increase precision, but they may also reduce sensitivity to recurring relationships that cross the boundaries through which those disciplines are organized. Investigating each condition independently can identify mechanisms contributing to its manifestations; comparison across conditions may reveal a different level of observation—substantially different challenges associated with overlapping patterns of functional burden.

Pathway specificity can therefore increase precision within an established explanatory framework while decreasing sensitivity to relationships that extend beyond that framework. This does not diminish the importance of pathway-specific investigation. It identifies a complementary role for discovery science, in which reliable observations can generate questions before the mechanisms organizing those observations are fully specified. Unexpected or recurring relationships may therefore be scientifically informative even when they cannot initially be assigned to a single pathway or disciplinary explanation.

Discovery does not relax the requirements of empirical science. Its value depends upon transforming observations into explicit hypotheses that can be distinguished from plausible alternatives. Platt (1964) described Strong Inference as a strategy in which multiple alternative hypotheses are formulated explicitly and observations or experiments are designed to distinguish among them. Rather than accumulating evidence consistent with a preferred explanation, the strategy emphasizes deriving predictions that discriminate among competing explanations and using the resulting evidence to constrain them. Discovery and Strong Inference are therefore complementary scientific strategies: discovery expands the range of relationships and explanations that can be considered, while Strong Inference constrains those explanations through empirical discrimination. The process is recursive: discovery generates hypotheses, discriminating tests constrain explanation, and unexpected findings create further opportunities for discovery.

The MRA emerged through such an exploratory process. It was not deduced from Polyvagal Theory, nor was it derived from the assumption of an unidentified anatomical pathway. It is proposed as an organizational hypothesis generated from recurring observations suggesting relationships among physiological regulation and adaptive function that may not be adequately represented when investigated only as independent effects.

Polyvagal Theory nevertheless provided an important organizational precedent. The Social Engagement System (Porges, 1998, 2003) was conceptualized as a functional system linking coordinated processes across anatomically distinct structures with different neural pathways and evolutionary origins. The MRA extends this organizational strategy beyond the functions incorporated within the Social Engagement System by asking whether a broader pattern of regulatory integration contributes to the organization of physiological state and adaptive function. Thus, the relationship between PVT and the MRA is one of conceptual and organizational expansion rather than theoretical deduction.

The constructs incorporated within this framework do not necessarily occupy equivalent levels of biological explanation or possess equivalent evidentiary status. Where an anatomical pathway can be identified directly, its structure, signaling, and mechanisms should be investigated at that level. Other constructs describe proposed functional relationships or emergent patterns of organization for which no single pathway provides an equivalent biological referent. The MRA is therefore neither an anatomical entity nor an alternative to pathway-specific explanation. It provides an organizational framework for investigating relationships among processes described at different biological and functional levels while preserving pathway-specific explanations wherever the evidence supports them. Its scientific value depends upon whether the proposed relationships generate empirically distinguishable propositions that can be supported, constrained, revised, or rejected.

Within this framework, signaling provides the means through which regulatory processes are differentially recruited and coordinated. Signaling therefore does more than transmit information among otherwise independent systems. By altering which processes participate and how they interact, signaling may functionally recruit components of the MRA into different regulatory configurations. Physiological state is proposed to reflect this coordinated organization rather than the activity of an isolated pathway or component.

The MRA is not intended as a fixed or exhaustive inventory of processes contributing to mammalian regulation. Its proposed components and relationships should expand, contract, or be revised as research identifies additional processes participating in system-level regulation. Inclusion within the proposed architecture is functional rather than determined by anatomical location: a process becomes relevant to the MRA to the extent that evidence indicates that its recruitment participates in relationships consequential for physiological state and adaptive function. Both the participating processes and their proposed relationships therefore remain empirically open to refinement.

This formulation adds an organizational level of inquiry without replacing pathway-specific explanation. Different initiating conditions may engage different pathways and proximal mechanisms while nevertheless having consequences that overlap at the level of physiological and adaptive function. Such overlap provides an empirical basis for asking whether pathway-specific effects influence broader regulatory organization; it does not establish a shared mechanism or common pathology.

One implication concerns the relationship between physiological regulation and adaptive function. Individuals may possess capacities for social engagement, learning, regulation, and recovery while varying substantially in their availability for recruitment and expression. Following substantial challenge, trauma, or illness, capacities that were previously available may become difficult to access without necessarily having been lost. This observation raises the possibility that prevailing physiological conditions influence the availability of existing capacities rather than simply determining whether those capacities are present or absent. This state-dependent availability of existing adaptive capacities is termed *Accessibility* and is developed formally in Section 5.

This reasoning identifies a complementary empirical pattern through which the organizational hypothesis can be interrogated. If apparently separate functional burdens are influenced by changes in broader regulatory organization, interventions that alter physiological regulation may be accompanied by improvement across multiple aspects of adaptive function without independently targeting each manifestation. Like the recurring burden illustrated in Figure 1, such multidomain improvement constitutes an observation requiring explanation rather than confirmation of a particular mechanism or architecture.

The empirical problem can therefore be approached from two complementary directions. Different challenges may be associated with overlapping patterns of functional burden, while interventions entering through different biological pathways may be associated with overlapping patterns of adaptive change. The first observation contributed to the discovery process through which the hypothesis of a broader regulatory architecture emerged. The second provides an opportunity to interrogate that hypothesis.

### 1.2 Evolutionary evidence as an independent constraint

Once an organizational relationship has been hypothesized from observation, a different scientific question follows: Is the proposed organization consistent with the evolutionary history and biological organization of mammals? Comparative and evolutionary biology provide an independent means of addressing this question. Evolutionary reasoning therefore does not provide the *a priori* premise from which the MRA is deduced. Rather, it provides evidence capable of constraining a discovery-generated organizational hypothesis.

Within this evolutionary inquiry, mammalian synapomorphies provide identifiable empirical anchors. In phylogenetic terminology, synapomorphies are shared derived characteristics. Mammalian synapomorphies refer here to identified shared derived characteristics associated with the mammalian lineage. The framework does not assume that the complete array of relevant mammalian synapomorphies has been identified, nor that the MRA can be derived from an exhaustive inventory of them. Instead, identified synapomorphies provide a basis for asking whether mammalian evolution was accompanied by changes in the functional relationships among regulatory processes.

The evolutionary proposition therefore concerns organization rather than the evolutionary novelty of every participating component. The relevant question is whether identified derived features associated with mammalian evolution were accompanied by changes in the functional relationships among evolutionarily older regulatory processes.

Evolutionary evidence consequently serves a different scientific function from the observations through which the MRA was initially generated. Recurring patterns of functional burden motivated the organizational hypothesis; patterns of adaptive change provide opportunities to interrogate it; and comparative evolutionary evidence provides an independent means of constraining its biological plausibility. These sources of evidence need not occupy the same level of biological explanation to contribute to evaluation of the framework.

The Introduction therefore returns to the empirical problem illustrated in Figure 1. Different challenges may produce overlapping patterns of functional burden without sharing a common pathology or proximal mechanism. The MRA proposes that this recurring organization warrants investigation rather than assuming either that each manifestation is independent or that recurrence establishes a common mechanism. Its scientific value depends upon whether the organizational hypothesis generates empirically distinguishable predictions and survives comparison with alternative explanations.

Section 2 develops this evolutionary constraint by examining how identified mammalian synapomorphies and comparative observations inform hypotheses concerning changes in the functional relationships among regulatory processes.

## 2 Evolutionary and scientific foundations of the mammalian regulatory architecture

Mammalian synapomorphies provide identifiable points of evolutionary change from which hypotheses concerning changes in regulatory organization can be examined. Darwin (1859) recognition that biological structures acquire meaning through their evolutionary history and adaptive function provides an early foundation for this approach. Hennig (1966) later formalized the concept of synapomorphy as a shared derived characteristic identifying evolutionary relationships among groups of organisms. Synapomorphies therefore identify characteristics that emerged within particular lineages and were inherited by their descendants. For the present argument, these identifiable evolutionary changes provide empirical points from which to examine whether relationships among regulatory processes also changed.

An important distinction follows from this comparative perspective. The presence of similar behaviors, physiological processes, or adaptive functions in different vertebrate groups does not necessarily imply that those functions are organized through identical neural or physiological mechanisms. Homologous structures and processes may participate in different functional organizations across lineages, while similar functions may emerge independently in response to comparable adaptive demands. Thus, the evolutionary question is not whether individual regulatory, communicative, caregiving, or social functions occur exclusively in mammals. It is whether mammalian evolution introduced derived features that altered the functional relationships among evolutionarily conserved processes.

When applied to mammals, identified synapomorphies and associated developmental characteristics invite this broader organizational question. Mammalian synapomorphies include features such as lactation and middle-ear specializations, while nursing and prolonged developmental dependency illustrate functional and developmental demands associated with mammalian reproduction and caregiving. These features did not emerge as isolated adaptations. They occurred within an organism confronting interdependent demands involving physiological regulation, reproduction, nursing, prolonged dependency, caregiving, communication, and increasing opportunities for social interaction. Dependency creates regulatory demands. Nursing requires proximity. Proximity requires communication and coordination. Prolonged developmental dependency increases the consequences of caregiving, reciprocal signaling, and the regulation of physiological state within relationships. Their relevance to the MRA therefore lies not simply in their presence as mammalian characteristics, but in whether their emergence was accompanied by changes in the organization through which conserved neural and physiological processes support adaptive function.

The importance of organization as a biological principle was articulated explicitly in the neurological writings of Jackson (1884). Jackson (1884) emphasized that nervous-system function cannot be understood solely by identifying individual structures or pathways. Function emerges through hierarchical and distributed relationships among neural systems, with newer organizational features modifying rather than simply replacing older ones. This principle is particularly relevant to evolutionary neuroscience because conserved structures may persist while the functional organizations in which they participate change as newer features emerge and become integrated with them. Evolutionary continuity of a structure therefore does not imply continuity of all of its functional relationships.

Jackson (1884) organizational principle addresses how evolutionary change may alter relationships among conserved and newer neural features. Dobzhansky (1973) adds a complementary adaptive perspective by emphasizing that biological traits acquire significance through their contribution to adaptation and the functioning of the organism. Evolutionary innovations derive their biological significance not solely from their status as derived characteristics, but also from the adaptive relationships into which they become incorporated. For the present framework, the relevant question is therefore not simply which characteristics distinguish mammals. It is whether derived mammalian features changed the conditions under which conserved regulatory processes operated and, through their functional integration, contributed to patterns of regulation supporting mammalian adaptation. Taken together, Jackson’s organizational principle and Dobzhansky’s adaptive perspective support a common implication: evolutionary significance may reside in changed relationships among biological processes as well as in the appearance of individual derived characteristics.

Developmental biology provides another source of evidence concerning these relationships. The present framework does not rely on classical recapitulation theory or assume that ontogeny reproduces phylogeny. Embryological development nevertheless provides a natural experiment through which biological systems are assembled, differentiated, and integrated. Developmental sequences can identify relationships among emerging structures and reveal how initially distinguishable processes become incorporated into increasingly differentiated patterns of regulation. Developmental evidence therefore complements comparative anatomy: comparative observations identify continuity and change across lineages, while development provides evidence concerning the differentiation and organization of biological processes within the organism.

This developmental perspective is particularly informative for understanding the organization of the mammalian vagus. The vagus is not a unitary pathway but a conduit containing biologically differentiated pathways associated with distinct physiological functions. Comparative anatomy, embryology, developmental neurobiology, neurophysiology, and psychophysiology provided converging observations supporting this interpretation before the internal organization of the human vagus could be directly characterized. Embryological investigations demonstrated differences in the developmental origins, maturation, and myelination of vagal fibers, while comparative observations identified conserved organizational principles together with mammalian specializations. These observations provided the biological foundation for proposing that vagal regulation is functionally differentiated rather than uniform.

Recent advances in human vagal anatomy provide an opportunity to investigate this proposition more directly. The Human Vagus Nerve Atlas (Zanos et al., 2026) demonstrates a reproducible internal fascicular organization of the human vagus and provides an anatomical framework for investigating how that organization relates to differentiated neural populations. These observations are consistent with the foundational Polyvagal Theory proposition that the vagus should not be conceptualized as a homogeneous pathway, but they do not test the more specific hypothesis that its internal organization reflects functionally differentiated pathways with distinguishable brainstem origins, fiber characteristics, peripheral targets, and physiological functions. The Atlas was designed to characterize fascicular organization rather than to establish these anatomical and functional relationships. This limitation reflects a broader asymmetry in the development of the relevant sciences. Theoretical, physiological, neurophysiological, and clinical observations—including rapidly developing applications of neuromodulation—have generated increasingly specific hypotheses about differentiated vagal function, while the anatomical evidence required to evaluate the corresponding organization within the human vagus has developed more slowly. Functional observations cannot resolve this anatomical gap. Hypotheses concerning the internal organization of the human vagus ultimately require direct anatomical evidence and therefore the engagement of anatomists and anatomical methods capable of identifying differentiated neural populations and relating them to fascicular organization, brainstem origins, fiber characteristics, and peripheral targets. The Human Vagus Nerve Atlas represents an important advance because it begins to provide the anatomical framework within which these hypotheses can now be investigated directly.

The limitation is therefore not that neuroanatomy is irrelevant to Polyvagal Theory, but that the relevant anatomical hypothesis has not yet been directly tested. Polyvagal Theory formulated this hypothesis from converging observations across comparative anatomy, embryology and developmental neurobiology, neurophysiology, and psychophysiology at a time when the internal organization of the human vagus was not directly accessible. Current anatomical observations establish internal organization and are consistent with biological differentiation, but fascicular organization alone cannot identify functional identity. Conversely, the absence of evidence linking fascicles to their central origins, fiber characteristics, peripheral targets, and physiological functions cannot constitute evidence against relationships that have not yet been investigated. Neuroanatomy can adjudicate this proposition, but doing so requires testing the anatomical hypothesis it actually specifies. The empirical task is therefore to determine whether and how these features are related within the human vagus. Although the Human Vagus Nerve Atlas does not directly link the anatomical organization of the peripheral vagus to its brainstem nuclei of origin, the methods developed to construct the Atlas make it increasingly feasible to investigate directly relationships that have largely been inferred from converging comparative, anatomical, developmental, and physiological observations.

Within this framework, particular attention has been directed toward vagal pathways associated with the nucleus ambiguus and the dorsal motor nucleus of the vagus. In mammals, Polyvagal Theory proposed a specific functional association between the myelinated cardioinhibitory pathway originating in the nucleus ambiguus and neural regulation of the striated muscles of the face and head involved in vocalization, ingestion, listening, and facial expression. Neither vocalization nor listening is proposed as a mammalian peculiarity (Porges, 2023). Birds and other non-mammalian vertebrates use acoustic signaling extensively in social communication and parenting, and birdsong provides a well-established example of convergent evolution in vocal learning and communication (Bolhuis et al., 2010). These examples demonstrate that similar communicative functions can emerge in different vertebrate lineages without establishing that the underlying neural and regulatory relationships are organized in the same way.

The mammalian proposition therefore concerns the organization of relationships among these processes, not their first appearance in mammals. Specifically, it concerns the proposed functional organization linking cardiovagal regulation with auditory processing and branchiomotor control of the face and head within the regulatory demands associated with nursing, proximity, caregiving, and co-regulation. What requires evaluation is this proposed relationship among distinguishable processes, not the evolutionary uniqueness of each process or adaptive function.

These proposed mammalian relationships provided the neuroanatomical foundation for the construct of the ventral vagal complex and, subsequently, the Social Engagement System within Polyvagal Theory. The ventral vagal complex is a functional construct and should not be interpreted as an anatomical synonym for the rhombencephalon or as incorporating all cardiovascular, respiratory, branchiomotor, and sensory networks within a single vagal structure. It refers more specifically to the proposed functional association between the myelinated cardioinhibitory pathway originating in the nucleus ambiguus and the regulation of striated muscles of the face and head. Cardiovascular-respiratory networks, branchiomotor pathways, and cranial and spinal sensory afferents remain anatomically distinguishable while participating in broader patterns of functional regulation.

Comparative evidence establishes evolutionary continuity in many of these components. Cardioinhibitory neurons intermingle with branchiomotor neurons in the amphibian nucleus ambiguus, and cranial motor organization supporting orofacial function is present in reptiles and other non-mammalian vertebrates (Matesz and Székely, 1996; Székely and Matesz, 1988; Taylor et al., 2022). These findings are incompatible with any claim that the constituent structures or functions first appeared in mammals; neither Polyvagal Theory nor the MRA makes such a claim.

More broadly, non-mammalian vertebrates necessarily integrate physiological, sensory, motor, and behavioral processes in support of adaptive function. The comparative question posed by the MRA is therefore not whether functional integration exists outside mammals. It is whether relationships among conserved regulatory processes were reorganized as they became integrated with derived mammalian features and with the regulatory demands associated with mammalian reproduction, development, and caregiving. Comparative evidence does not by itself establish that these organizational relationships differ across lineages, just as similarity in individual structures or functions does not establish that the relationships are identical. The evolutionary proposition concerns the proposed pattern of organization among conserved and derived regulatory features and therefore requires evidence directed toward that proposition.

The Social Engagement System introduced an important organizational step in the development of this argument (Porges, 1998). It did not define a functional system by a single anatomical structure, neural pathway, or evolutionary origin. It is neither localized to a discrete brain region nor equivalent to the whole brain. Rather, it proposed functional relationships among anatomically and physiologically distinguishable processes through which behavioral signaling and physiological state could become coordinated. Social communication and functional organizations supporting social behavior occur outside mammals. The mammalian proposition concerns a hypothesized pattern of functional organization linking distinguishable regulatory processes in relation to mammalian specializations involving nursing, prolonged dependency, caregiving, and co-regulation.

The significance of the Social Engagement System for the MRA is therefore organizational rather than anatomical. The MRA extends the organizational principle illustrated by the Social Engagement System rather than enlarging the Social Engagement System itself. The relevant continuity is the strategy of asking how biologically distinguishable processes may acquire additional functional significance through their coordinated relationships. The MRA extends this question to broader relationships among physiological regulation and adaptive function while preserving the anatomical and physiological distinctions among the processes participating in those relationships. It is therefore neither deduced from Polyvagal Theory nor conceptualized as a larger anatomical system into which the Social Engagement System is incorporated.

This conceptual progression—from identifiable evolutionary and biological observations, through proposed functional organization within the Social Engagement System, to the broader organizational hypothesis of the MRA—is summarized in Figure 2.

**Figure 2:**
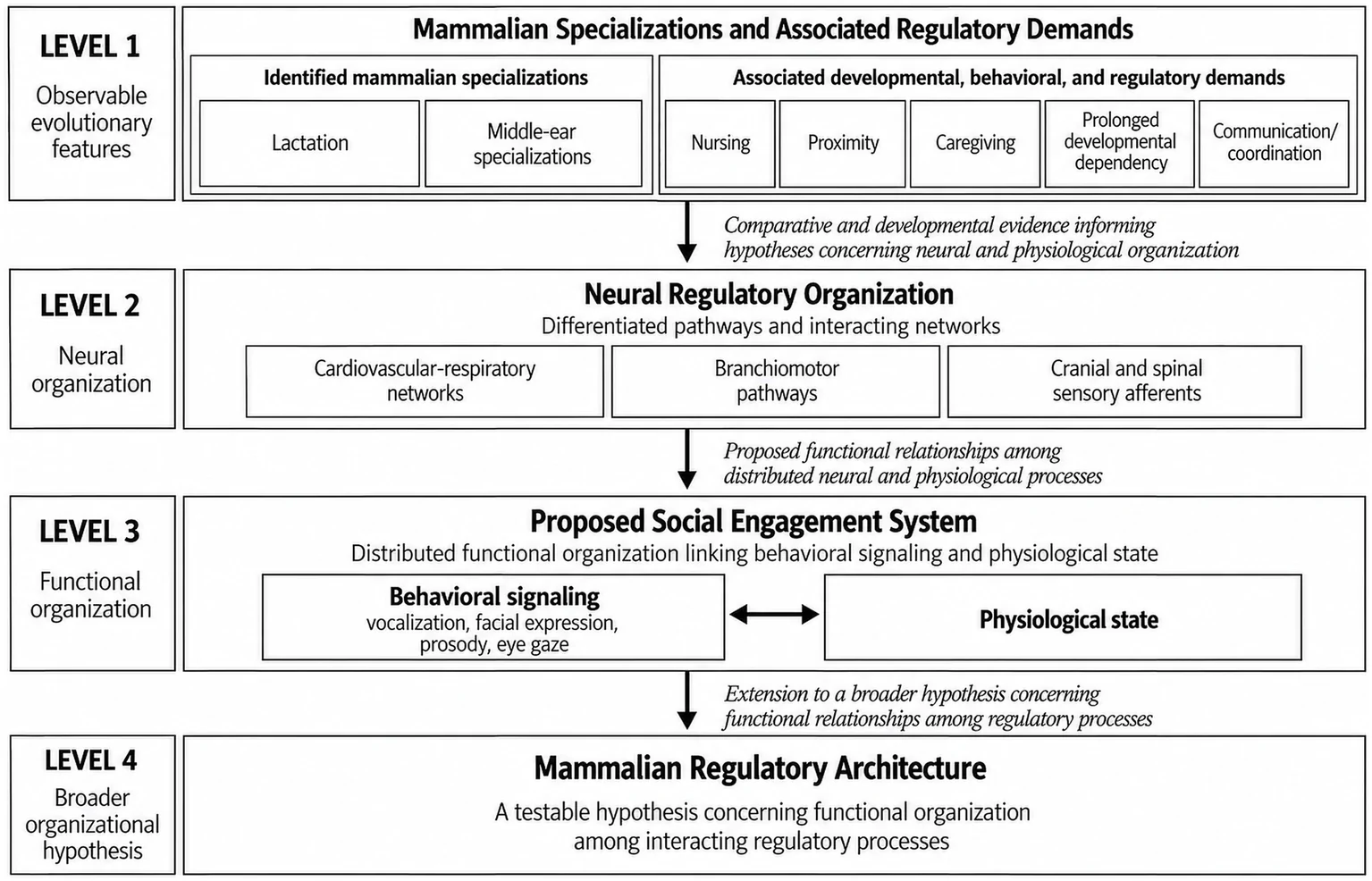
From mammalian specializations to mammalian regulatory architecture. Conceptual progression from identifiable mammalian specializations and associated regulatory demands to neural and functional organization and the broader Mammalian Regulatory Architecture. The levels represent distinct biological propositions and evidentiary status rather than an anatomical hierarchy. Arrows indicate relationships informing conceptual development, not a unidirectional causal sequence.

Figure 2 distinguishes levels of biological claim as well as stages in the conceptual development of the MRA. Identified mammalian characteristics and distinguishable neural pathways have direct comparative, anatomical, developmental, or physiological referents. The Social Engagement System represents a proposed functional organization among distinguishable processes, while the MRA extends the organizational question to broader relationships among regulatory processes. These levels are neither an anatomical hierarchy nor a causal sequence. Evidence establishing the presence, evolutionary history, or physiological function of a component does not by itself establish the functional organization in which that component participates; conversely, an organizational hypothesis cannot substitute for anatomical or physiological evidence when a pathway-specific claim is made. Figure 2 therefore leads to a methodological question: How should propositions formulated at different levels of biological organization be evaluated?

### 2.1 Levels of analysis, representational Fidelity, and empirical evaluation

The conceptual progression summarized in Figure 2 traverses distinguishable levels of biological explanation. Observable characteristics, anatomical structures, neural pathways, physiological functions, functional systems, and broader regulatory organization are related, but they are not interchangeable levels of analysis. Evidence relevant to one level does not necessarily adjudicate propositions formulated at another. The presence of a structure in another vertebrate lineage, for example, does not determine whether relationships among that structure and other regulatory processes were reorganized during mammalian evolution. Similarly, evidence concerning the function of an individual neural pathway does not by itself establish or reject a proposition concerning its participation within a broader functional organization.

These levels identify different scientific questions rather than a hierarchy in which organizational explanations supersede anatomical or pathway-specific explanations. A pathway-specific proposition requires pathway-specific evidence; a proposition concerning functional organization requires evidence capable of evaluating relationships among the processes included in that proposition. The level at which a claim is formulated therefore constrains the evidence required to test it.

Biology and physiology have long accommodated questions formulated at different levels of organization. Mayr (1961) distinguished proximate from evolutionary explanation without treating them as competing accounts, while Bernard (1957) concept of the *milieu intérieur*, Cannon (1932) formulation of homeostasis, and Hess (1958) demonstration of patterned central regulation established the importance of coordinated physiological and neural regulation beyond the activity of individual organs or outputs. Wiener (1948) formulation of cybernetics emphasized regulation, feedback, and communication among interacting processes, while von Bertalanffy’s (1968) General System Theory emphasized properties of organized systems that cannot be adequately characterized from their components considered independently. These traditions do not diminish the importance of anatomy or pathway-specific mechanisms; they establish the scientific legitimacy of asking complementary questions at the level of functional organization.

Neuromodulation makes the relationship between these levels experimentally consequential. An intervention necessarily engages the organism through identifiable anatomical, sensory, neural, or physiological pathways. Characterizing those pathways is essential for determining how an intervention initially engages the nervous system and for testing mechanisms attributed to that route of engagement. Organizational analysis asks a complementary question: What is the functional significance of that pathway engagement within the broader regulatory organization? Effects initiated through a specific pathway may become distributed across multiple regulatory processes, while interventions entering through different pathways may produce overlapping functional outcomes. Identifying the pathway of initial engagement therefore does not by itself explain how that engagement participates within the broader regulatory organization through which distributed physiological or functional effects may become expressed.

Empirical evaluation consequently requires fidelity at three related levels. *Information fidelity* concerns the accuracy with which observations, evidence, and sources are represented. *Representational fidelity* concerns whether the proposition being evaluated corresponds to the proposition actually advanced. *Inferential fidelity* concerns whether conclusions drawn from evidence are warranted by that evidence and whether the evidence addresses the proposition at the level at which it is formulated. Together, these requirements provide a basis for evaluating claims across different levels of biological explanation without allowing evidence appropriate to one level to substitute automatically for evidence required at another.

Representational fidelity is especially important when evaluating theoretical propositions. When a proposition is replaced by a reformulation that the theory did not make, evidence addressing that reformulation cannot be treated as evidence addressing the original claim. In this circumstance, the reformulation functions as a strawman regardless of whether the misrepresentation was intentional. Inferential fidelity imposes a complementary requirement. Evidence concerning anatomy cannot automatically adjudicate a proposition concerning functional organization, just as evidence concerning a single pathway cannot by itself establish or reject a proposition concerning relationships among multiple regulatory processes. Conversely, an organizational proposition should not be used to make pathway-specific claims that have not been independently established. Evidence inconsistent with a proposed anatomical, physiological, evolutionary, or organizational relationship should constrain, revise, or reject that relationship; evidence addressing a different proposition cannot perform that function.

The Grossman legacy of criticism of Polyvagal Theory provides a concrete example of why these requirements matter. Grossman and Taylor (2007) characterized several propositions of Polyvagal Theory in ways identified as inaccurate in a contemporaneous response (Porges, 2007). Related attributions nevertheless persisted in the literature despite those published clarifications (Porges, 2023) and reappear in Grossman et al. (2026). A systematic historical audit (Porges, 2026) documents recurrent problems of information fidelity, representational fidelity, and inferential fidelity involving claims concerning anatomical organization, RSA, respiratory–cardiac coupling across vertebrate lineages, and evolutionary interpretation. The relevance of these documented problems extends beyond the individual criticisms: when conclusions based on failures of scientific fidelity persist in the scientific literature, they may become treated as constituting an unresolved scientific controversy.

Those consequences become particularly important when evidence concerning individual components, pathways, or physiological phenomena is treated as sufficient to adjudicate propositions concerning biological organization. Evidence for evolutionary continuity at the component level may then be assumed to challenge differentiation at the organizational level, even though organizational differentiation has not itself been tested. Relationships among processes, their temporal coordination, and the functional capacities supported by their organization may consequently become marginalized as independent objects of inquiry.

The scientific cost of this inferential error extends beyond false conclusions about a particular theory. Science advances through the generation of viable questions and testable hypotheses as well as through increasingly precise answers. Scientific disciplines necessarily develop specialized methods, constructs, and levels of analysis that provide rigor and cumulative knowledge. This specialization can generate productive tension when observations require questions that cross established explanatory boundaries. The tension becomes costly, however, when those boundaries constrain which questions are considered scientifically legitimate. When conclusions generated at the level of components or pathways are assumed to settle questions at an organizational level, potentially informative hypotheses may not simply remain unanswered; they may cease to be asked. The cost is borne in the generation of knowledge and, ultimately, in the capacity to formulate and solve biological and clinical problems.

The Grossman legacy is therefore relevant to the MRA at two levels: documented failures of scientific fidelity produced false conclusions about Polyvagal Theory, while the persistence of those conclusions as an assumed scientific controversy illustrates the broader cost of allowing evidence at one level of biological organization to foreclose questions at another level it never tested. The organizational perspective evident in the original formulation of Polyvagal Theory provides a concrete example of what may otherwise be obscured.

Richter and Spyer (1990) described a common cardiorespiratory oscillator involving central neural processes coordinating respiratory and cardiac regulation. This organizational observation was incorporated into the original formulation of Polyvagal Theory (Porges, 1995) and subsequently elaborated in relation to the neural regulation of respiratory–cardiac interactions (Porges, 2007). Its significance for the present argument lies not simply in the presence of respiratory–cardiac coupling, but in the central neural organization through which respiratory and cardiac regulation are coordinated. This organization involves interactions between the NTS and NA and distinguishes the common cardiorespiratory oscillator from the DMNX.

This distinction is critical. Demonstrating respiration-related variation in heart rate across vertebrate lineages documents the occurrence of a physiological phenomenon across those lineages; it does not establish continuity of the neural organization coordinating that phenomenon. Comparative evidence documents evolutionary differentiation in the distribution of cardiac vagal preganglionic neurons, with increasing representation in ventrolateral brainstem regions and, in mammals, a major cardioinhibitory influence originating in the nucleus ambiguus (Taylor, 1994; Taylor et al., 1999; Porges, 1995). Moreover, cardiac vagal neurons within the nucleus ambiguus are dynamically modulated across the respiratory cycle, providing direct electrophysiological evidence that central respiratory processes pattern cardiac vagal efferent activity (Gilbey et al., 1984). The comparative question therefore concerns whether the neural organization coordinating respiratory and cardiac regulation has been conserved or reorganized, rather than whether vagal pathways or respiratory–cardiac interactions themselves are present. Evolutionary differentiation may reside in relationships among conserved and derived components even when many of the components themselves have deep ancestral histories.

Predator–prey differences provide another illustration at the level of adaptive function. Predator and prey mammals share many autonomic and brainstem regulatory components, yet those components may be coordinated differently under challenge. Profound bradycardia may participate in defensive immobilization in prey mammals, whereas comparable chronotropic responses have not been observed in predator mammals such as dogs and cats despite other cardiovascular effects of dorsal motor nucleus stimulation (Porges, 2023). These observations do not establish a pathway from behavior alone; rather, they illustrate how shared regulatory components may participate in different organizations supporting different species-typical adaptive capacities.

The criticisms of Polyvagal Theory advanced by Grossman et al., 2026 and colleagues concerning myelinated vagal pathways, respiratory sinus arrhythmia, and defensive responses are inaccurate because they evaluate propositions different from those formulated by the theory. The presence of myelinated cardiac vagal fibers or nucleus ambiguus-associated cardioinhibitory neurons outside mammals does not invalidate Polyvagal Theory, because the theory does not claim that these individual components are anatomically exclusive to mammals. Such observations establish evolutionary antecedents outside the mammalian lineage but do not determine whether their developmental trajectories, central coordination with respiration and cranial motor processes, or functional relationships were reorganized in mammals. The relevant comparative proposition concerns evolutionary changes in the organization and functional relationships among these components, not the first appearance of an isolated pathway. Thus, demonstrating the presence of individual components in non-mammalian vertebrates does not test, and therefore cannot invalidate, the organizational proposition advanced by Polyvagal Theory (Grossman and Taylor, 2007; Grossman et al., 2026; Porges, 2007, 2023, 2026).

Within Polyvagal Theory, respiratory sinus arrhythmia (RSA) in mammals has been interpreted as a peripheral expression of respiratory-linked cardiac vagal regulation associated with the nucleus ambiguus. RSA is neither a measure of all vagal activity nor a direct readout of a broader Social Engagement System or the MRA. Its amplitude can also be affected by respiratory and measurement conditions; interpretation therefore requires attention to respiration, species, development, and the neural pathways generating the observed rhythm. Respiratory-linked heart-period variation across vertebrates documents a shared physiological phenomenon, while the organization of the neural processes generating it remains a separate comparative question. Evidence showing that the specified mammalian RSA pattern does not depend on the proposed neural organization would constrain that pathway-specific proposition (Grossman and Taylor, 2007; Porges, 2007, 2023).

Polyvagal Theory has also proposed that pathways originating in the dorsal motor nucleus of the vagus may contribute to profound cardiac slowing during defensive immobilization in some species and developmental conditions (Porges, 2023). This proposition does not imply that all immobility or behavioral shutdown is caused by dorsal vagal activity, that bradycardia is present in every defensive response, or that behavior alone identifies a vagal source nucleus. The dorsal motor nucleus also participates in visceral regulation, and its cardiovascular consequences may differ by species and context. Direct neural, cardiovascular, and behavioral measurements are needed to distinguish a proposed dorsal vagal contribution from nucleus ambiguus-associated and sympathetic influences. Findings inconsistent with the specified source, conditions, or predicted physiological response should revise that proposition rather than be assigned to a vagal pathway on the basis of behavior alone.

Mammalian synapomorphies provide the evolutionary context for these neurophysiological and functional observations. Derived mammalian characteristics occur within a lineage that also retains extensive ancestral continuity. Their existence therefore makes explicit why continuity of components cannot, by itself, determine continuity of organization. The scientific question is not whether mammals retained ancestral structures and physiological processes—they clearly did—but whether relationships among conserved and derived processes changed during mammalian evolution and what functional capacities emerged from those relationships. The Social Engagement System represents one formulation of this organizational question within Polyvagal Theory; the MRA extends the question to broader relationships among regulatory processes.

The Mammalian Regulatory Architecture provides a conceptual framework within which this organizational question can be formulated and empirically evaluated. It preserves the importance of pathway-specific neuroanatomy and neurophysiology while making relationships among conserved and derived regulatory processes an explicit object of biological investigation. The MRA does not require that the participating processes be uniquely mammalian; rather, it asks whether relationships among conserved and derived processes changed in consequential ways within the mammalian lineage and how those relationships contribute to adaptive function. Thus, evolutionary continuity and mammalian differentiation become complementary empirical questions rather than competing explanations.

Importantly, documenting failures of scientific fidelity that invalidate criticisms of Polyvagal Theory does not provide evidence for propositions formulated at another level. The Grossman legacy is relevant here because it illustrates how misrepresentations and inferential errors can persist when evidence at one level of biological organization is treated as adjudicating propositions at another level that was not tested. Invalidating those criticisms does not, however, provide evidentiary support for the MRA. The mammalian synapomorphies and the constructs developed through Polyvagal Theory and the Social Engagement System contributed to development of the MRA and constrain the biological plausibility of its organizational hypothesis, but they do not establish it. The MRA is subject to the same requirement of scientific fidelity: its propositions must be tested with evidence appropriate to the level at which they are formulated and reconsidered, refined, or rejected when that evidence does not support them. Recognizing organization as a distinct level of analysis therefore does not insulate the MRA from empirical evaluation; it preserves the scientific possibility of asking and testing organizational questions.

If mammalian evolution was accompanied by changes in the functional relationships among conserved and derived regulatory processes, the next question concerns the regulatory consequences of that reorganization. The relevant proposition is not simply that mammals possess particular structures or regulatory capacities, but that their organization altered how biologically consequential information could influence physiological regulation. Section 3 develops this proposition by examining safety as a regulatory property of the proposed Mammalian Regulatory Architecture and asking how information associated with conditions of safety may influence physiological regulation and the proposed integration of regulatory processes within the MRA.

## 3 Mammalian synapomorphy and the emergence of a regulatory architecture

### 3.1 Safety as a regulatory property of the mammalian architecture

The evolutionary perspective developed in Section 2 suggests that the biological significance of safety cannot be reduced to the absence of threat. Within the proposed Mammalian Regulatory Architecture, safety is relevant because information associated with conditions of safety may influence physiological regulation and thereby alter the functional capacities available to the organism (Porges, 2022). The mammalian significance proposed here does not depend on an exclusive mammalian capacity to detect or respond to safety. Rather, consistent with the organizational argument developed in Section 2, the relevant question concerns whether and how sensory, contextual, and relational information became integrated with regulatory processes supporting physiological state and adaptive function.

This distinction is illustrated by maternal social buffering in domestic chickens. The presence of a hen reduced her chicks’ behavioral response to an aversive stimulus (Edgar et al., 2015). Thus, social buffering and responsiveness to safety-associated social information occur outside the mammalian lineage. The observed response does not, by itself, establish the chicks’ subjective experience of safety. The mammalian proposition concerns the organization of regulatory relationships associated with mammalian specializations, rather than the first appearance of social buffering.

This formulation requires distinctions among several levels of analysis that are frequently treated as interchangeable. Objective environmental safety refers to properties of the environment that can be characterized independently of the individual’s response. Cues associated with safety are sensory, contextual, or relational features that may acquire biological significance through evolutionary history, development, or experience. Neuroception, as proposed within Polyvagal Theory, refers to neural processes through which features associated with risk or safety may influence autonomic regulation without requiring conscious awareness (Porges, 2003, 2004, 2022). Cognitive appraisal refers to conscious or cognitively mediated evaluation of circumstances, whereas feelings of safety refer to subjective experience. Physiological state refers to the dynamically organized physiological condition of the organism. Accessibility refers to the state-dependent availability of existing capacities. Functional outcomes refer to consequences expressed in behavior, cognition, social engagement, restoration, or other domains. These constructs may be related within the proposed architecture, but they are not interchangeable.

The relationships among these levels are not assumed to be one-to-one. An objectively safe environment need not produce a physiological state supporting feelings of safety, and subjective feelings of safety do not establish that an environment is objectively safe. Similarly, a cue associated with safety does not necessarily produce a predictable physiological response in every individual or context. Developmental history, prior experience, current physiological state, illness, and contextual conditions may alter the regulatory consequences of the same sensory or relational information (Porges, 2020, 2022). Neuroception therefore should not be inferred solely from the physiological or behavioral response that follows, just as subjective feeling or functional outcome should not be used retrospectively to classify an environment as safe. Maintaining these distinctions prevents circular reasoning and makes the proposed relationships independently available for empirical evaluation.

Within the MRA, information associated with safety is proposed to influence physiological organization and thereby the *Accessibility* of existing capacities. To the extent that such information contributes to a shift from physiological states organized around defense, capacities constrained within those states may become more available to support restoration, engagement, and adaptive function. These capacities are not produced by safety-associated information or created by physiological state, nor are they direct readouts of a particular pathway. Rather, physiological state is proposed to regulate their Accessibility and expression.

The feeling of safety provides a particularly relevant example of state-dependent Accessibility. An environment may be objectively safe and cognitively appraised as safe while a physiological state organized around defense limits access to the embodied experience of safety. Conversely, a physiological state supporting engagement and reduced defensive mobilization may increase the Accessibility of feelings of safety without establishing that the environment is objectively safe. Thus, objective safety, cognitive appraisal of safety, physiological state, and feeling safe represent related but distinguishable levels of analysis. The capacity to experience safety is not created by physiological state; rather, its Accessibility is proposed to be constrained or supported by physiological organization.

The organizational perspective evident in Polyvagal Theory provides a precedent for this formulation. Polyvagal Theory proposed that cues associated with safety may influence autonomic regulation through neural processes described as neuroception and through the Social Engagement System (Porges, 1998, 2003, 2004, 2007, 2022). The MRA extends this organizational perspective by situating autonomic and social-engagement processes within a broader regulatory architecture involving interacting sensory, communicative, neuroendocrine, immune, caregiving, and social processes. Safety is therefore treated not as a property of a particular cue, pathway, or autonomic component, but in terms of how information associated with safety participates in this broader regulatory organization.

This organizational formulation generates an empirical hypothesis: the regulatory influence of information associated with safety should be reflected in physiological organization and in its relationship to the *Accessibility* of existing capacities. Evidence that a stimulus is perceived as safe, that a person reports feeling safe, or that a single physiological variable changes following exposure to a safety-associated cue cannot by itself establish this organizational relationship. Evaluation therefore requires independently characterizing relevant levels—for example, properties of the environment or stimulus, physiological responses, subjective experience, and functional outcomes—and testing relationships among them without defining one from the occurrence of another. Evidence for the proposed organizational role of safety must therefore come from relationships among independently characterized levels rather than inference from any single stimulus, feeling, physiological response, or functional outcome. Figure 3 summarizes these proposed relationships within the broader architecture.

**Figure 3:**
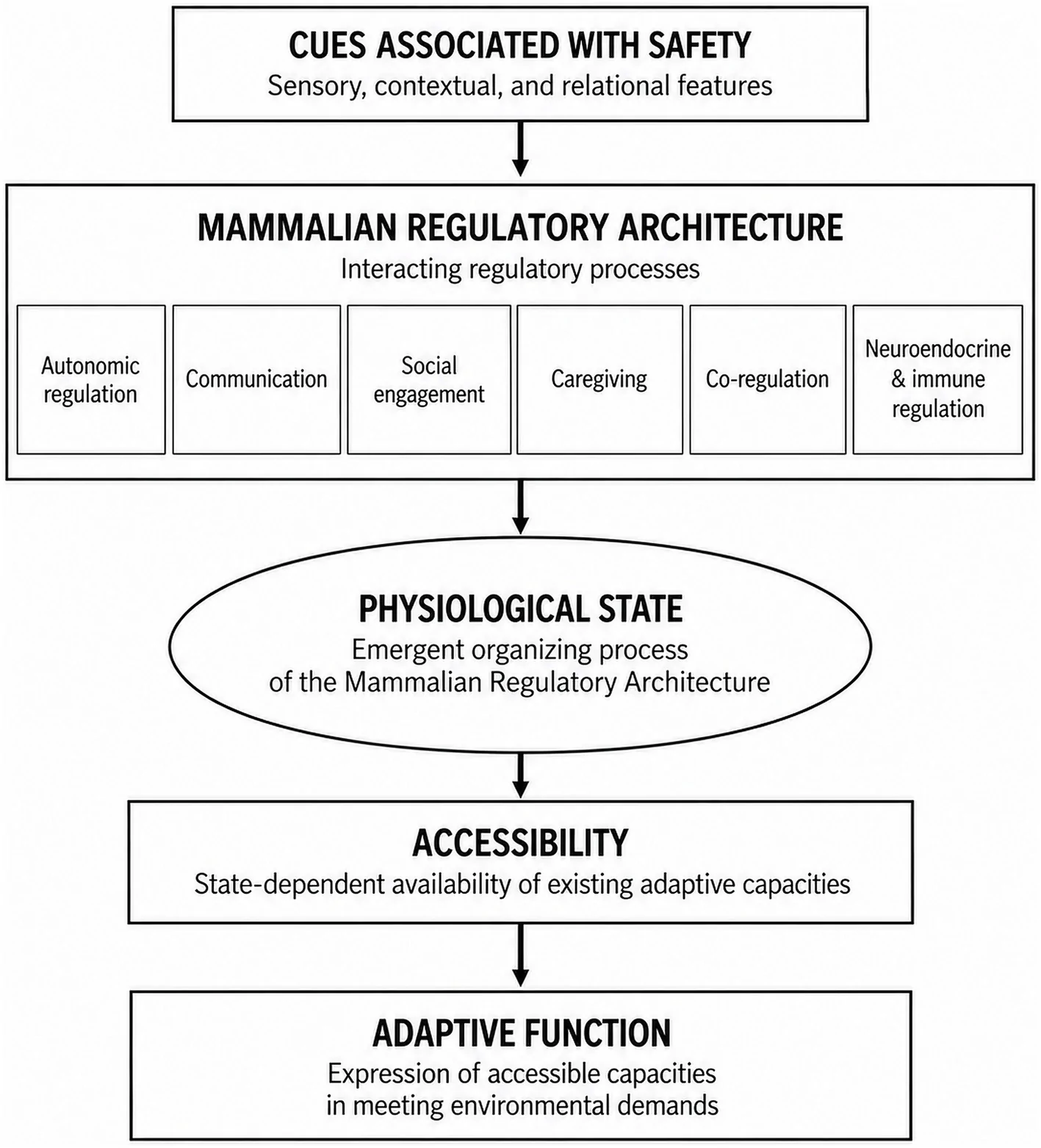
Safety-related cues, physiological state, and accessibility within the mammalian regulatory architecture. Cues associated with safety may engage the Mammalian Regulatory Architecture, contributing to physiological state and state-dependent Accessibility of adaptive capacities. Arrows represent proposed sequential functional contingencies, not a fixed causal sequence.

The regulatory significance attributed to safety consequently depends on integration. For information associated with safety to influence capacities across physiological, behavioral, communicative, and social domains, its regulatory consequences cannot be understood solely from the pathway through which the information initially enters the nervous system. The relevant question becomes how the effects of that information are coordinated across interacting regulatory processes. Section 3.2 develops this organizational question by examining integration as a defining feature of the Mammalian Regulatory Architecture.

### 3.2 Integration of regulatory systems

The defining feature of the proposed Mammalian Regulatory Architecture is not any individual structure, pathway, or regulatory process, but the functional integration of distinguishable processes whose relationships may contribute to physiological state and adaptive function.

The evolutionary basis for this proposition lies in the possibility that mammalian synapomorphies altered not only individual structures or functions but also the regulatory conditions under which conserved biological processes operated. Lactation provides a particularly informative example. Milk production is a mammalian synapomorphy, but its regulatory significance extends beyond the mammary gland. Nursing requires coordination among maternal physiology, offspring ingestion, proximity, sensory communication, and caregiving behavior. The prolonged developmental dependency characteristic of mammalian offspring further increases the functional consequences of these relationships. The relevant evolutionary question is therefore not whether ingestion, communication, caregiving, or physiological regulation originated in mammals, but whether derived mammalian features changed how these conserved processes became functionally related.

This formulation distinguishes evolutionary novelty from functional integration. Homologous components do not require identical functional relationships across vertebrate lineages, just as analogous functions do not require identical mechanisms. Non-mammalian vertebrates integrate sensory, physiological, motor, communicative, and behavioral processes in support of adaptive function. The mammalian proposition is more specific: derived features associated with mammalian reproduction and development may have altered relationships among conserved regulatory processes and thereby changed the organization through which physiological state, caregiving, communication, and adaptive function became coordinated.

The differentiation of oxytocin and vasopressin signaling provides a complementary evolutionary observation. These closely related neuropeptide systems have deep evolutionary histories, while their differentiation in mammals became associated with functions including parturition, lactation, maternal behavior, fluid regulation, and social processes. Their relevance to the MRA is not that either peptide constitutes a mammalian regulatory architecture or uniquely mediates mammalian sociality. Rather, their differentiation provides another potential point of evolutionary change at which reproductive, physiological, caregiving, and social regulatory demands may have become differently organized. As with other mammalian synapomorphies, the observation generates a question about functional relationships rather than establishing the architecture itself.

The proposed MRA therefore concerns integration of conserved and derived features rather than replacement of older regulatory systems by newer ones. Evolutionarily conserved autonomic, sensory, visceral, endocrine, and motor processes remain biologically distinguishable, while mammalian specializations may have altered the functional relationships in which they participate. Integration does not imply fusion into a single anatomical system or common mechanism. It refers to dynamic relationships through which activity in one regulatory domain may alter the conditions under which other processes operate. These relationships may change with internal conditions, environmental demands, and relational information, and particular processes may become more or less influential as conditions change, without requiring a fixed wiring diagram or central controller.

Evidence for the participating components does not, however, establish their functional integration. Demonstrating a neural pathway, endocrine signal, immune process, sensory mechanism, or behavioral function establishes evidence at the level of that component; the MRA makes the additional proposition that relationships among such processes are functionally consequential. Mammalian synapomorphies therefore provide empirical anchors for asking how these relationships may have changed during evolution, while comparative and developmental evidence constrain the plausibility of the proposed organization. Neither establishes the MRA. The architecture becomes scientifically informative to the extent that its proposed relationships can be translated into empirically distinguishable predictions.

This formulation raises the next question: what does functional integration mean biologically, and how can it be observed without reducing the architecture either to its component pathways or to an unconstrained systems-level description? Section 3.3 addresses this question by defining the functional boundaries of the MRA and specifying the role of physiological state within its proposed organization.

### 3.3 Functional organization and empirical evaluation of the MRA

The boundaries of the Mammalian Regulatory Architecture are functional rather than anatomical. The MRA is not proposed as a discrete structure or as an exhaustive inventory of biological processes within a mammal. Rather, it refers to proposed functional relationships among distinguishable regulatory processes that contribute to physiological organization and adaptive function. A process therefore becomes relevant to the proposed architecture through its participation in functionally consequential relationships, not by membership in a particular anatomical system.

Processes and relationships incorporated within the MRA may be described at different levels of biological explanation and evaluated through different forms of evidence. Where an anatomical pathway can be identified directly, its structure, signaling, and mechanisms should be investigated at that level. Other constructs describe functional relationships or emergent patterns of organization for which no single pathway provides an equivalent biological referent. The MRA therefore adds an organizational level of inquiry without replacing pathway-specific explanation.

Reciprocity is central to this organizational proposition. Physiological state refers to the emergent organization expressed through the coordinated activity and interactions of multiple regulatory processes. It is not positioned as an independent controller within the architecture. Rather, the prevailing organization provides the physiological context within which participating processes continue to operate and interact. Physiological state therefore refers to the organization of these relationships rather than to any single pathway, mechanism, or physiological variable.

Physiological state is therefore a biological construct to be characterized through specified features of physiological organization, not a predetermined composite score or necessarily a latent statistical variable. A composite or latent-variable model may be useful for a particular empirical question, but its validity would depend on the processes and relationships it represents. Physiological state is also not synonymous with stress, arousal, affective valence, autonomic balance, or subjective wellbeing. Each may describe an aspect or consequence of regulation without characterizing the relationships and temporal coordination among the processes relevant to a particular hypothesis. To avoid circularity, physiological organization must be characterized from physiological observations independently of the behavioral, subjective, or adaptive outcomes it is proposed to explain. The relevant processes, relationships, and time scales must be specified in advance for the question under study rather than inferred retrospectively from a favorable outcome.

Signaling provides one means through which this organization can be dynamically altered. In this context, a signal reflects a change in information that can become an effective input to regulatory processes. The source of that change may arise within or outside the body and is not restricted to a particular sensory or physiological domain. Sensory information becomes an effective input to neural regulatory circuits through transduction and afferent signaling, involving the molecular and electrophysiological processes through which those circuits operate. Afferent signals conveying changes in internal physiological conditions are often viewed as neural feedback mechanisms involved in homeostatic regulation. Within the MRA, however, the functional significance of signaling is broader: inputs arising within or outside the body may alter which regulatory processes are recruited and how they interact, thereby changing the functional organization expressed as physiological state. Although signals enter through distinguishable pathways, their regulatory consequences need not remain confined to the domain through which the information entered. The resulting response therefore reflects not only the pathway through which information enters but also how that input interacts with the broader regulatory architecture. The functional significance of a signal within the MRA can therefore be evaluated by determining whether a defined change in input is accompanied by measurable changes in the recruitment, coordination, or relationships among regulatory processes. A signal may be detectable without producing such organizational effects, and the same signal may have different regulatory consequences depending on the prevailing physiological state. The architecture is therefore dynamic rather than fixed, while its proposed components and relationships remain open to empirical refinement.

Ingestion provides a concrete example of this organizational perspective. Eating recruits identifiable sensory, motor, autonomic, and visceral pathways, yet pathway identification alone does not specify why ingestion may support affiliation, co-regulation, and physiological conditions compatible with digestion in one context while becoming aversive, defensive, or socially isolating in another. Disorders of eating are informative because sensory and visceral experience, physiological regulation, behavior, and social participation may interact without requiring a common pathway or mechanism. This formulation does not propose disruption of the MRA as a unitary cause of eating disorders; rather, ingestion provides an empirical context in which reciprocal relationships among distinguishable processes can be investigated.

Figure 4 generalizes this organizational principle across representative regulatory processes. The paired relationships emphasize that physiological organization is dynamic and reciprocal rather than a unidirectional causal sequence. As signals arising within and outside the body alter the recruitment and coordination of regulatory processes, these relationships may be continuously adjusted in relation to changing conditions. Homeodynamic retuning describes this continuing adjustment of regulatory organization.

**Figure 4:**
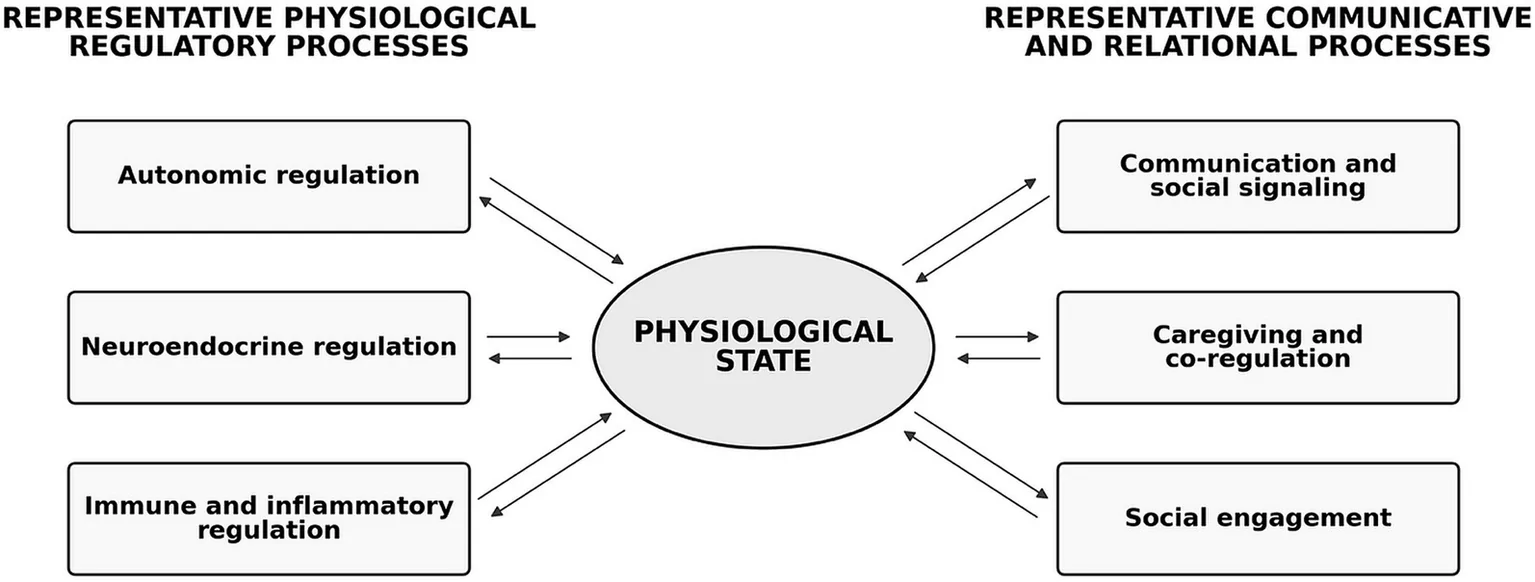
Reciprocal organization within the proposed mammalian regulatory architecture. Representative regulatory, communicative, and relational processes contribute to physiological state and operate within the physiological context it provides. These reciprocal relationships provide a proposed basis for homeodynamic retuning as conditions change.

The reciprocal organization represented in Figure 4 overlaps with established descriptions of integrated biological regulation but is not defined by that overlap. The central autonomic network describes distributed and reciprocally interconnected central neural structures, including hypothalamic and brainstem regions, involved in processing visceral information and coordinating autonomic regulation. The MRA does not replace this neural-network description. Rather, it proposes a broader level of functional organization in which central autonomic networks participate in relationships with other physiological processes and with ingestive, communicative, behavioral, caregiving, and relational functions. The difference is therefore one of level and scope: the central autonomic network characterizes dynamic neural organization participating in autonomic regulation, whereas the MRA addresses proposed functional relationships extending across neural, physiological, and adaptive processes.

The MRA also intersects with established concepts of biological regulation. Homeostasis describes processes supporting stability of the internal milieu, allostasis emphasizes adaptive adjustment in relation to anticipated and changing demands, and homeodynamics emphasizes the continuously changing organization through which physiological regulation is sustained. Predictive regulation addresses anticipation of demands and adjustment of responses, while systems biology provides conceptual and methodological approaches for studying interactions among components. These perspectives establish that dynamic and interactive regulation is not itself distinctive to the MRA. The MRA addresses a more specific organizational proposition: relationships among regulatory processes may constitute a functionally consequential level of organization linking physiological state with sensory, ingestive, communicative, relational, and adaptive functions. Homeodynamic retuning therefore describes the ongoing adjustment of relationships within the proposed architecture; it does not define the architecture itself.

This proposition has direct empirical implications. If the MRA concerns functional relationships among regulatory processes, those relationships themselves become targets of measurement. Evaluation does not require a comprehensive measure of the MRA. Theoretically selected relationships among independently measured processes can test specific propositions derived from the architecture. The relevant question is therefore not only whether individual physiological variables change, but whether their relationships and temporal organization change systematically with context, challenge, recovery, or intervention.

These relationships can be investigated using multivariable physiological measures that preserve temporal information and permit coordination among independently measured processes to be characterized. Importantly, the temporal patterning of peripheral physiological signals may contain information about the neural processes regulating them, while relationships among signals may reveal organizational properties not captured by considering the component measures independently. Relational measures need not identify a common neural mechanism to provide evidence concerning physiological organization. Section 6 develops these methodological principles in the context of neuromodulation and considers how dynamic physiological organization can be characterized following engagement through distinct biological routes.

## 4 The portal model: neural and sensory pathways into the mammalian regulatory architecture

Neuromodulation has conventionally been conceptualized in relation to identifiable neural targets and pathways through which interventions influence nervous system function. This pathway-centered approach is essential for determining how an intervention initially engages the nervous system and for identifying neural and neurochemical mechanisms through which particular effects may be generated. However, the expanding range of physiological, cognitive, behavioral, and functional outcomes associated with neuromodulatory interventions raises an additional question: Does the pathway through which an intervention enters the nervous system provide a sufficient level of explanation for the breadth of its effects?

For the purposes of this framework, a neuromodulatory intervention is an intentionally applied procedure designed to engage an identifiable neural, sensory, or physiological route in order to alter neural or physiological regulation. This classification concerns the intervention and its proposed route of engagement; it does not presume that the intended state-regulating effect occurred. Incidental environmental stimulation, spontaneous social interaction, ordinary emotional experience, and unprompted physiological fluctuation are therefore not classified as neuromodulation merely because they can influence physiological state. Structured acoustic, somatosensory, respiratory, or direct neural interventions can be evaluated under this definition when their route and intended regulatory effect are specified.

The Mammalian Regulatory Architecture extends the explanatory focus from the pathway initially engaged to the broader regulatory organization potentially influenced through that engagement. Within this framework, a pathway identifies an anatomical or physiological route through which an intervention engages the nervous system. A mechanism identifies a process through which a particular effect is generated. A portal describes the functional significance of a pathway or regulatory process through which signaling gains access to the MRA. Pathway, mechanism, and portal are therefore related but non-equivalent constructs. A portal is not a separate anatomical structure, neural pathway, or mechanism of action. Rather, it identifies the functional significance of a route of access to the broader regulatory architecture. The Portal Model therefore expands rather than replaces pathway-centered and mechanistic explanation.

The Portal Model distinguishes two complementary levels of regulation: portals govern input, whereas the architecture governs the response profile. A portal specifies and constrains how an intervention or biologically consequential signal gains access to the MRA, including what information enters the architecture and how it is initially represented. Once the architecture is engaged, however, the resulting response is not determined by the portal or by any single pathway. The response profile is an organizational construct, reflecting the pattern of physiological and functional consequences that emerges from relationships among regulatory processes within the MRA. It therefore reflects both the physiological state in which the input is received and how signaling initiated through the portal is integrated across the architecture.

Feedback is fundamental to this architecture-level regulation and, more generally, to the organization of living systems (Wiener, 1948; von Bertalanffy, 1968). Signaling entering through a portal can influence regulatory processes beyond the domain in which the signal originated, while the resulting changes can feed back upon the organization governing subsequent responses. The response profile therefore cannot be reduced to the pathway of entry. It reflects the organization of relationships among processes participating in the MRA. Thus, the portal constrains input, whereas the architecture governs how that input is integrated and expressed across physiological, behavioral, cognitive, communicative, neuroendocrine, immune, and other functional domains.

Because response profile is an organizational rather than pathway-specific construct, portal and response cannot be mapped one-to-one. The same portal may be associated with different response profiles when the physiological state or broader regulatory organization of the recipient differs, whereas distinct portals may produce convergent response profiles when their engagement recruits overlapping features of the MRA. Portal-specific effects may nevertheless remain because the characteristics of the input and the pathways initially engaged are not erased by architecture-level integration.

Mammalian evolution gave this general feedback organization additional functional significance. Feedback itself is phylogenetically ancient; the proposed mammalian specialization concerns, in part, how conserved signaling and feedback processes became integrated with derived regulatory features supporting nursing, caregiving, prolonged developmental dependency, reciprocal communication, and co-regulation (Porges, 2021). Behavior, especially social behavior, could thereby become a potent source of regulatory signaling. Behavior generated by one individual can provide sensory information that is detected and transduced through afferent pathways in another, while the recipient’s regulatory architecture governs the resulting response profile. If engagement through these pathways influences broader regulatory organization, they acquire functional significance as biological portals. The recipient’s resulting behavior may, in turn, provide sensory information to the other individual. Co-regulation thus provides a functional example of reciprocal signaling across individuals and illustrates how mammalian-derived regulatory features expanded the functional significance of feedback by enabling social behavior to participate in physiological regulation.

Sensory and mechanosensory engagement provide concrete examples of this principle. Touch, pressure, vibration, and acoustic stimulation can generate afferent signaling through routes that vary with the site and characteristics of stimulation. Somatosensory information from the face and head may be conveyed through cranial sensory afferents, whereas somatosensory information from the trunk and extremities is conveyed predominantly through spinal afferent pathways (Kandel et al., 2021). Cranial sensory afferents may also provide sensory input relevant to the neural regulation of the Social Engagement System (Porges, 1998, 2001). A single sensory event may recruit more than one afferent route. When such stimulation is intentionally structured to influence neural or physiological regulation, it can be evaluated as neuromodulation and its functional significance as a biological portal tested empirically.

Touch illustrates a non-vagal mode of sensory engagement. Pleasant, gentle touch can recruit low-threshold unmyelinated cutaneous afferents implicated in affective touch (Löken et al., 2009), while pleasant deep pressure can engage additional mechanosensory processes (Case et al., 2021). Depending on the site and characteristics of stimulation, somatosensory signaling may enter through spinal or cranial sensory pathways, including trigeminal pathways, rather than through the vagus. These distinct afferent routes specify how sensory input enters the nervous system; whether their engagement influences broader physiological organization and adaptive function is a separate empirical question concerning their significance as biological portals.

Vagus nerve stimulation provides a particularly informative illustration because its initial pathway is relatively well characterized. Peripheral VNS is commonly interpreted as influencing central regulation primarily through vagal afferents projecting to the nucleus of the solitary tract (NTS) and associated central networks (Frasch and Porges, 2023; Staats et al., 2024). This afferent account is consistent with, but should be distinguished from, the differentiated efferent vagal pathways emphasized within Polyvagal Theory. Polyvagal Theory has described bidirectional communication in which sensory information from internal organs is conveyed through afferent pathways to brainstem regulatory structures, while motor pathways regulate the organs from the brain (Porges, 1993, 2001, 2003). Interoceptive afferent information may therefore influence physiological state, while functionally distinguishable efferent vagal pathways associated with the nucleus ambiguus and dorsal motor nucleus provide routes through which regulation may be expressed. Vagal afferent stimulation identifies the route through which VNS initially engages central regulatory processes and should not be conflated with direct recruitment of these differentiated efferent pathways. Rather, the afferent pathway may function as a portal through which broader state-regulatory organization is influenced, with consequences potentially expressed through differentiated vagal efferent pathways and other processes participating in the MRA.

The NTS illustrates the transition from pathway-centered to architecture-centered explanation. At the pathway level, the NTS is a major recipient of vagal afferent information. Through its extensive connections with brainstem, forebrain, autonomic, neuroendocrine, and other regulatory networks, however, activity reaching the NTS can influence processes extending beyond the initiating vagal afferent pathway. The NTS is therefore not the portal itself. Rather, it illustrates how activity entering through an identifiable pathway can become consequential for a broader regulatory organization. The transition from pathway to architecture occurs when the explanatory question shifts from how a signal is transmitted to how the consequences of that signaling participate in the organization of physiological state.

Pathway-specific mechanisms remain essential within this broader account. The inflammatory reflex, for example, provides an influential mechanistic framework for understanding how vagal signaling may influence inflammatory regulation (Tracey, 2002, 2007; Pavlov and Tracey, 2012). Inflammatory control is not exclusively vagal; sympathetic pathways, including the greater splanchnic nerves, also contribute to reflex regulation of inflammation (Martelli et al., 2014). These vagal and sympathetic mechanisms identify distinguishable biological processes through which particular effects may be generated and should not be collapsed into a single autonomic mechanism.

A demonstrated pathway-specific mechanism, however, need not explain the broader consequences of engagement through the same pathway. VNS has been applied or investigated across domains including depression, migraine and other forms of pain, gastrointestinal function and functional disorders, and cognitive functions including learning and memory. A mechanism demonstrated within one regulatory domain need not explain effects expressed across others. The architecture-level question is whether engagement through the vagal pathway influences physiological organization and, through that organization, functions expressed across multiple regulatory and behavioral domains. This proposition neither requires those outcomes to share a single mechanism nor replaces pathway-specific explanations.

Respiratory engagement further illustrates why a biological portal need not be reduced to a single pathway. Breathing practices alter respiratory timing and can modulate respiratory-linked cardiac vagal efferent activity, including the nucleus ambiguus-associated pathway indexed by RSA under appropriate conditions. This does not imply that every breathing practice selectively recruits or exercises that pathway. Breathing simultaneously recruits vagal pulmonary afferents and interacts with brainstem respiratory networks, thoracic mechanics, gas exchange, and baroreflex processes. The ventral vagal component therefore represents an identifiable pathway-specific contribution within a broader mode of respiratory engagement. The Portal Model accommodates this distributed engagement by asking whether these interacting processes influence physiological organization within the MRA rather than requiring respiratory effects to be assigned to a single pathway.

Structured auditory stimulation provides a particularly informative contrast with vagal stimulation. tcVNS engages the nervous system through a visceral afferent route, whereas structured auditory stimulation provides input through auditory-sensory routes initiated by structured acoustic information. Their initial modes of engagement and identified pathways are therefore substantially different. The Portal Model does not assume that either intervention is represented by a single afferent pathway; rather, each provides a distinguishable mode of input through which signaling may engage the broader regulatory architecture.

In the randomized Long COVID study described in Section 6.1, Sonic Augmentation Technology (SAT), which provides structured auditory stimulation, and tcVNS were associated with similar patterns of multidomain improvement despite their markedly different modes of engagement. This provides an empirical example of outcome convergence. Within the proposed framework, SAT and tcVNS are hypothesized to engage the MRA through distinct biological portals. Their convergent outcomes do not establish physiological convergence, identical changes in physiological organization, or a shared neural or neurochemical mechanism. Observed outcomes represent only one expression of the broader organizational response profile, and convergence in those outcomes does not establish convergence in the regulatory organization generating them. Rather, outcome convergence generates an architecture-level prediction requiring physiological evidence: if these distinct modes of engagement function as biological portals to the MRA, their different inputs should be associated with convergence in aspects of physiological organization while potentially retaining portal- or pathway-specific physiological signatures (see Figure 5).

**Figure 5:**
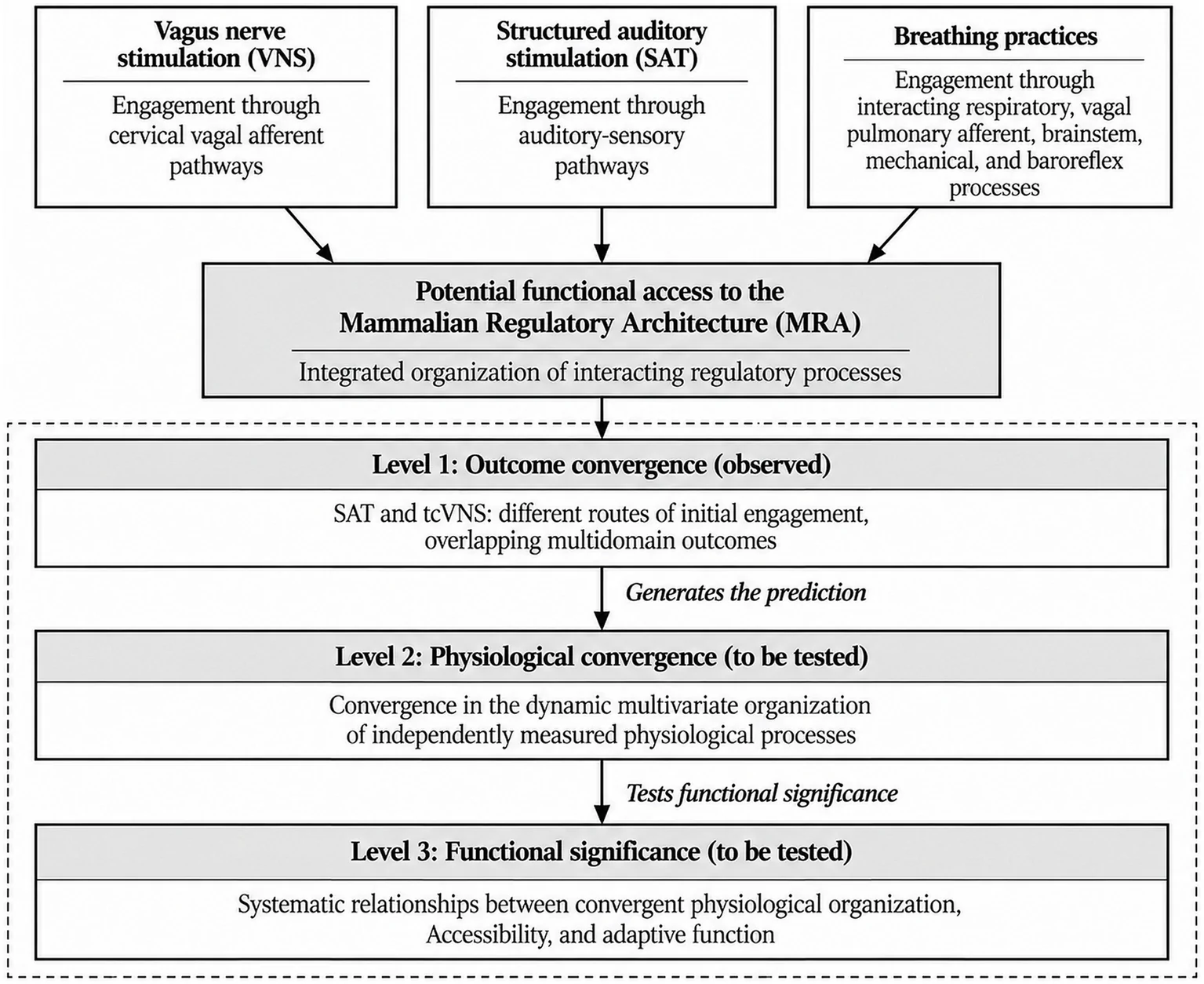
Distinct modes of engagement and evaluation of the portal model. Distinct neural, sensory, and physiological routes may provide functional access to the Mammalian Regulatory Architecture. Observed outcome convergence generates testable predictions concerning physiological convergence and its relationship to Accessibility and adaptive function.

The Portal Model can therefore be evaluated at three empirically distinguishable levels. Outcome convergence identifies overlapping adaptive outcomes following interventions that engage the organism through different routes but does not establish physiological convergence, portal function, or a common mechanism. Physiological convergence requires evidence that engagement through different routes produces convergence in aspects of physiological organization rather than merely similar changes in individual physiological variables. Functional significance requires determining whether those organizational features are systematically related to changes in Accessibility and adaptive function. The empirical requirements for establishing physiological convergence are developed more fully in Section 6.

Consistent with the Strong Inference strategy introduced in Section 1, alternative explanations for apparent outcome convergence must also be evaluated. Similar outcomes may reflect expectancy, provider interaction, relaxation, changes in breathing or arousal, regression to the mean, characteristics of the outcome measures, natural recovery, publication bias in the broader intervention literature, or other nonspecific influences. Their relevance will vary with intervention and study design and should be evaluated empirically rather than assumed either to account for convergence or to be excluded by it. Their plausibility further underscores why outcome convergence cannot itself establish convergence in physiological organization or a common mechanism.

Table 1 summarizes representative modes of engagement, their proposed function as biological portals, and the evidence required to evaluate their relationship to the MRA.

**Table 1:** Representative modes of access to the mammalian regulatory architecture.

Mode of engagement	Route and proposed portal	Evidence relevant to the MRA
Vagus nerve stimulation (VNS)	Direct vagal stimulation recruits a relatively well-characterized afferent pathway to the NTS and associated central networks. Within the proposed framework, the recruited pathway may function as a biological portal through which vagal stimulation gains functional access to regulatory processes participating in the MRA.	Multidomain outcomes and physiological effects have been reported. Whether engagement produces broader convergent changes in multivariate physiological organization requires direct testing.
Structured auditory stimulation (SAT)	Structured acoustic information initially engages auditory-sensory pathways. Within the proposed framework, this auditory engagement may function as a biological portal through which SAT gains functional access to regulatory processes participating in the MRA. This proposition does not require prior specification of the complete intervening neural or neurochemical sequence.	Multidomain outcomes and physiological effects have been observed. Broader multivariate physiological convergence has not been established.
Breathing practices	Respiratory engagement recruits respiratory-linked cardiac vagal regulation together with pulmonary afferent, brainstem respiratory, mechanical, gas-exchange, and baroreflex processes. These interacting processes may collectively provide functional access to the MRA rather than constituting a single pathway.	Respiratory and cardiac measures, including respiratory sinus arrhythmia, index selected processes. Broader changes in physiological organization and their relationships with Accessibility and adaptive function remain testable predictions.

A biological portal describes the functional significance of engagement through a pathway or regulatory process as access to the MRA; it does not designate a separate anatomical structure or mechanism.

## 5 Physiological state and accessibility: linking neuromodulation to adaptive function

The Portal Model developed in the preceding section identifies how engagement through a neural, sensory, or other regulatory pathway may provide access to processes participating in the Mammalian Regulatory Architecture. This raises the next question: How might changes in physiological organization become consequential for adaptive function? Addressing this question requires distinguishing the presence of an adaptive capacity from its availability for recruitment and expression.

Functions readily expressed under one physiological condition may become difficult to recruit under another. A person may think clearly, communicate spontaneously, engage socially, or mobilize effectively under one condition and find these same responses difficult under another. Comparable state-dependent changes are evident in visceral regulation and restorative processes. Importantly, these changes may occur without evidence that the underlying capacities have been lost. A failure to express a function therefore does not necessarily indicate the absence or loss of the capacity supporting that function. What may change is whether an existing capacity is available for recruitment under the prevailing physiological conditions.

This distinction directs attention to physiological state. As developed in the preceding sections, physiological state emerges from the coordinated and reciprocal activity of interacting regulatory processes within the Mammalian Regulatory Architecture rather than from a single physiological variable or pathway. Physiological organization refers to the dynamic pattern of relationships among these regulatory processes, including their coordination, reciprocal influence, and temporal patterning. Physiological state is an emergent property expressed through this organization. Changes in physiological organization may therefore alter the conditions under which existing capacities are recruited and expressed.

This relationship becomes particularly apparent under challenge. A physiological state organized to support defense may increase Accessibility to protective responses while making other functions more difficult to recruit. As physiological organization changes, capacities that had become less accessible may become available again for expression. Trauma provides a particularly informative example. Survivors may know what they wish to say or do while being unable to recruit responses that are readily available under other physiological conditions. The capacity may therefore remain present even when its expression is constrained. Such observations make explicit why the presence of a capacity and its momentary availability cannot be treated as equivalent.

Accessibility describes the state-dependent availability of an existing capacity. Accessibility does not refer to the acquisition, creation, or loss of the capacity itself. It refers to whether an existing capacity is available for recruitment and expression within the prevailing physiological state. A capacity may therefore remain present while its Accessibility changes.

Accessibility is also capacity specific. A change in physiological state does not imply uniform change across functions. The same physiological state may facilitate some responses while constraining others. A physiological state that increases Accessibility to social communication, cognitive flexibility, or visceral and restorative functions may simultaneously reduce Accessibility to protective mobilization. Under a differently organized physiological state, this pattern may be reversed. Physiological state should therefore not be represented as a global continuum extending from impaired to optimal function. Different physiological states may facilitate different adaptive functions.

Accessibility must also be distinguished from observable function. Capacity refers to an existing functional potential. Accessibility refers to whether that capacity is available for recruitment within the prevailing physiological state. Observable function reflects the expression of that capacity under particular conditions. These constructs are related, but they are not interchangeable. Observable function is influenced not only by physiological state but also by learning, experience, motivation, context, and task demands. Consequently, a change in observable performance does not by itself establish either a change in the underlying capacity or a change in its Accessibility.

Because Accessibility is inferred rather than directly observed, its empirical evaluation requires establishing that a capacity is present and can be expressed under some conditions, and then determining whether its availability for expression changes systematically in relation to independently characterized changes in physiological organization. Observable function can therefore provide evidence relevant to Accessibility, but observable function cannot itself define Accessibility. Experimental designs must distinguish state-dependent changes in the availability of an established capacity from changes attributable to learning, experience, motivation, context, or task demands. Accessibility is thus more specific than a general designation of readiness, resilience, or behavioral flexibility: it concerns the state-dependent availability of an identified capacity under specified physiological and task conditions.

Accessibility is not defined by a fixed statistical role. In a prospective study, a change in Accessibility could be tested as a mediator if independently measured physiological reorganization precedes a change in the availability of an established capacity and that change helps account for subsequent adaptive function. Alternatively, the physiological organization or Accessibility present before an intervention could be tested as a moderator of its effects. These are distinct empirical models, not properties established by the definition of Accessibility. Reciprocal relationships may also occur as behavior and experience alter physiological conditions. Neither mediation nor moderation can be inferred from improvement in performance alone; each requires measurements and temporal or experimental comparisons appropriate to the proposed relationship.

An empirical example illustrating the importance of this distinction is provided by research using the Listening Project Protocol, an acoustic intervention developed for laboratory research and subsequently developed into the Safe and Sound Protocol (SSP). In autistic participants, five sessions of the intervention were followed by significant improvements on the Filtered Words and Competing Words subtests of the SCAN, measures requiring central auditory processing. Following the intervention, performance on these measures was no longer significantly different from that of the typically developing comparison group (Porges et al., 2013). Improved auditory-processing performance was accompanied by a significant increase in baseline respiratory sinus arrhythmia (RSA), an index of cardiac vagal regulation relevant to physiological state. Thus, the study documented parallel changes in physiological regulation and performance on central auditory-processing functions that had not been directly trained.

Because the study was preliminary and did not include a randomized control condition, these findings do not establish that the change in physiological regulation caused the improvement in auditory processing or demonstrate increased Accessibility. They do, however, illustrate the empirical question made explicit by the Accessibility construct: when an independently measured change in physiological regulation accompanies improved performance on an untrained function, might the change reflect altered Accessibility to an existing capacity rather than acquisition of a new capacity?

The Accessibility hypothesis makes this interpretation experimentally testable. Improved performance following an intervention does not itself demonstrate increased Accessibility. Rather, the interpretation requires evidence that the relevant capacity was already present and that changes in its availability for expression are systematically related to independently characterized changes in physiological organization. The empirical question therefore shifts from whether performance improved to whether an established capacity became more available for recruitment as physiological organization changed.

Figure 6 illustrates how the same existing capacities may remain present across physiological states while differing in their Accessibility and observable expression. The pattern is capacity specific: a physiological state that increases Accessibility to some capacities may reduce Accessibility to others. Thus, the figure does not represent movement toward a universally optimal physiological state.

**Figure 6:**
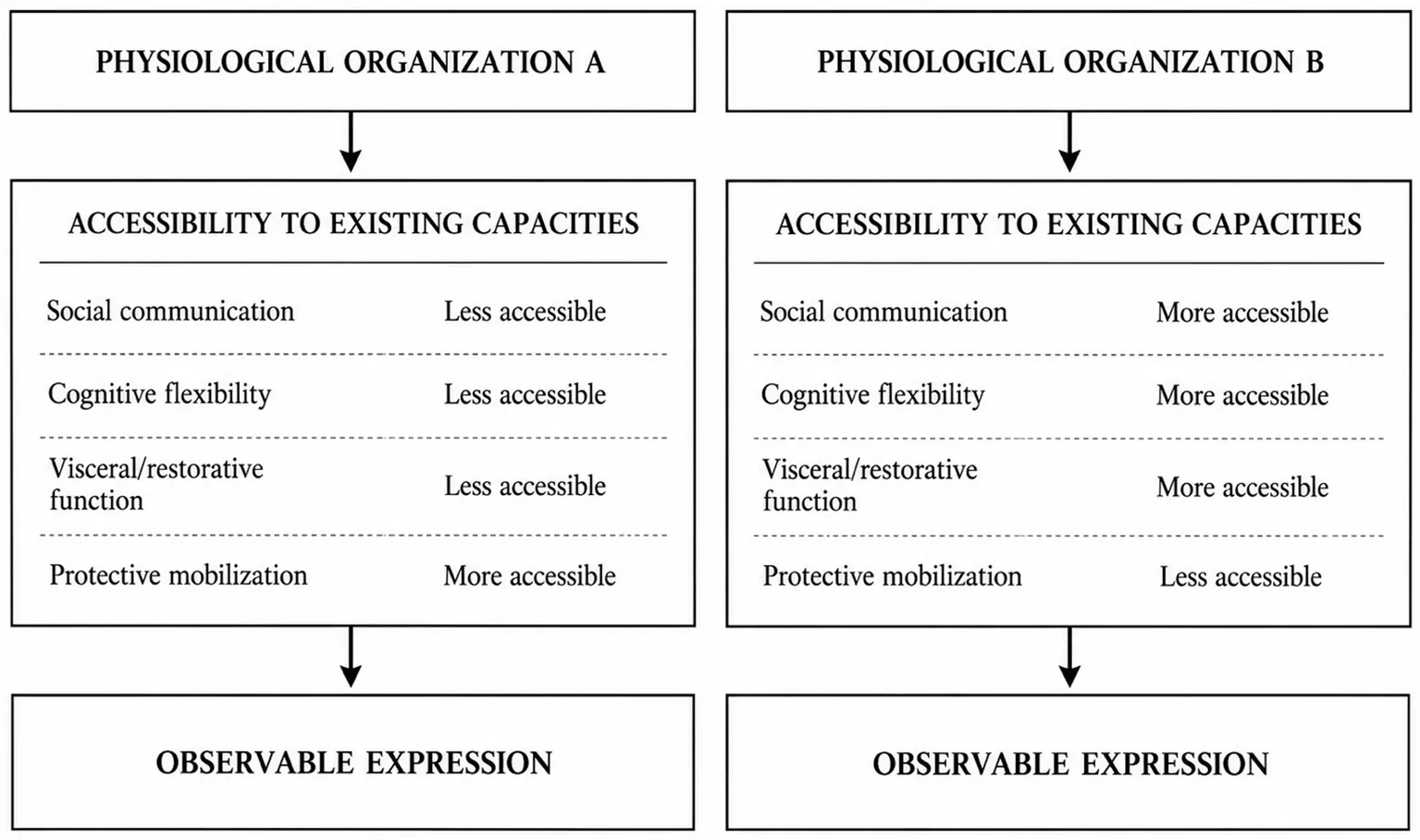
Physiological state and accessibility to existing capacities. Existing capacities may remain present across physiological states while differing in their state-dependent Accessibility and observable expression. Examples are hypothetical.

Psychophysiological and neurophysiological research provides a means of evaluating this proposition. Functional changes can be examined in relation to independently measured changes in physiological regulation rather than inferred from behavior alone. Because physiological state is conceptualized as an emergent property of the organization of interacting regulatory processes, it cannot be established by identifying change in any single physiological process. Individual physiological measures provide empirical access to selected processes participating in physiological organization, while relationships among these measures provide evidence relevant to their coordination and temporal patterning. Behavioral and functional measures, in turn, provide evidence concerning the expression of particular capacities and, under appropriately controlled conditions, their availability for recruitment. The relevant evidence therefore lies in systematic relationships between independently characterized changes in physiological organization and changes in the availability and expression of capacities known to be present.

Neuromodulation extends this logic from observation to experimental perturbation. Engagement through a biological portal provides an opportunity to determine whether influencing regulatory processes participating in the MRA changes physiological organization and whether those changes are systematically related to the Accessibility of established capacities. The route of initial engagement, changes in physiological organization, and changes in the availability and expression of adaptive capacities can therefore be characterized independently rather than inferred from one another.

This formulation generates a direct empirical question: If engagement through a biological portal changes physiological organization, are those changes accompanied by predictable changes in the Accessibility and expression of existing adaptive capacities? An adaptive outcome alone cannot answer this question. Characterizing the route of engagement identifies how an intervention initially engages the nervous system; multivariable physiological measurement evaluates whether relationships and temporal coordination among participating regulatory processes change; and functional measurement evaluates whether established capacities become differentially available for recruitment and expression. When distinct biological portals produce overlapping outcomes, this same logic provides an additional test of functional significance: whether convergence in physiological organization is systematically related to convergence in Accessibility and adaptive function. This relationship provides the functional bridge from the Portal Model to the architecture-centered analysis of neuromodulation developed in the following section.

## 6 Reframing neuromodulation through the mammalian regulatory architecture

The preceding sections distinguish the pathway through which an intervention initially engages the nervous system from its functional significance as a biological portal and identify physiological state and Accessibility as constructs for evaluating the broader consequences of that engagement. These distinctions shift the scientific question from whether an intervention affects a particular pathway or outcome to whether engagement through that pathway alters physiological organization in ways that become consequential for Accessibility and adaptive function. Pathway-centered analyses remain essential for identifying routes of initial engagement and pathway-specific mechanisms; the MRA adds an architecture-centered level of analysis concerned with how interacting physiological regulatory processes are organized and how changes in that organization become consequential for physiological state, Accessibility, and adaptive function.

This distinction becomes particularly relevant when neuromodulatory effects extend beyond functions readily attributed to the initially engaged pathway or to a single established mechanism. Multidomain effects do not demonstrate a common mechanism. They generate an organizational question: does engagement through a specific biological route alter relationships among physiological regulatory processes participating in the MRA, producing a broader response profile that becomes consequential across multiple functions?

Answering this question changes what must be measured. Pathway-centered analyses characterize the route initially engaged and its associated mechanisms or physiological signatures. Architecture-centered analyses require independent characterization of physiological organization as a dynamic pattern of relationships among physiological regulatory processes, together with independent assessment of the Accessibility and expression of adaptive capacities. Because physiological state is an emergent property of physiological organization, change in a single physiological process cannot establish an architecture-level effect, just as an adaptive outcome alone does not establish Accessibility. Evidence for a change in physiological organization therefore resides in systematic changes in relationships and temporal coordination among independently characterized physiological processes, not simply in the number of physiological variables that change. Establishing the functional significance of that change additionally requires determining whether it is systematically related to changes in the Accessibility and expression of established capacities.

The relationships summarized in Figure 7 should not be interpreted as a unidirectional progression from intervention to outcome. Portal engagement may influence physiological organization and physiological state, while the prevailing physiological state influences the conditions under which existing adaptive capacities become accessible. Changes in physiological organization may also alter subsequent responses to portal engagement. The hypothesis therefore concerns systematic relationships among distinguishable processes and functional levels within an interactive regulatory architecture rather than a fixed causal chain.

**Figure 7:**
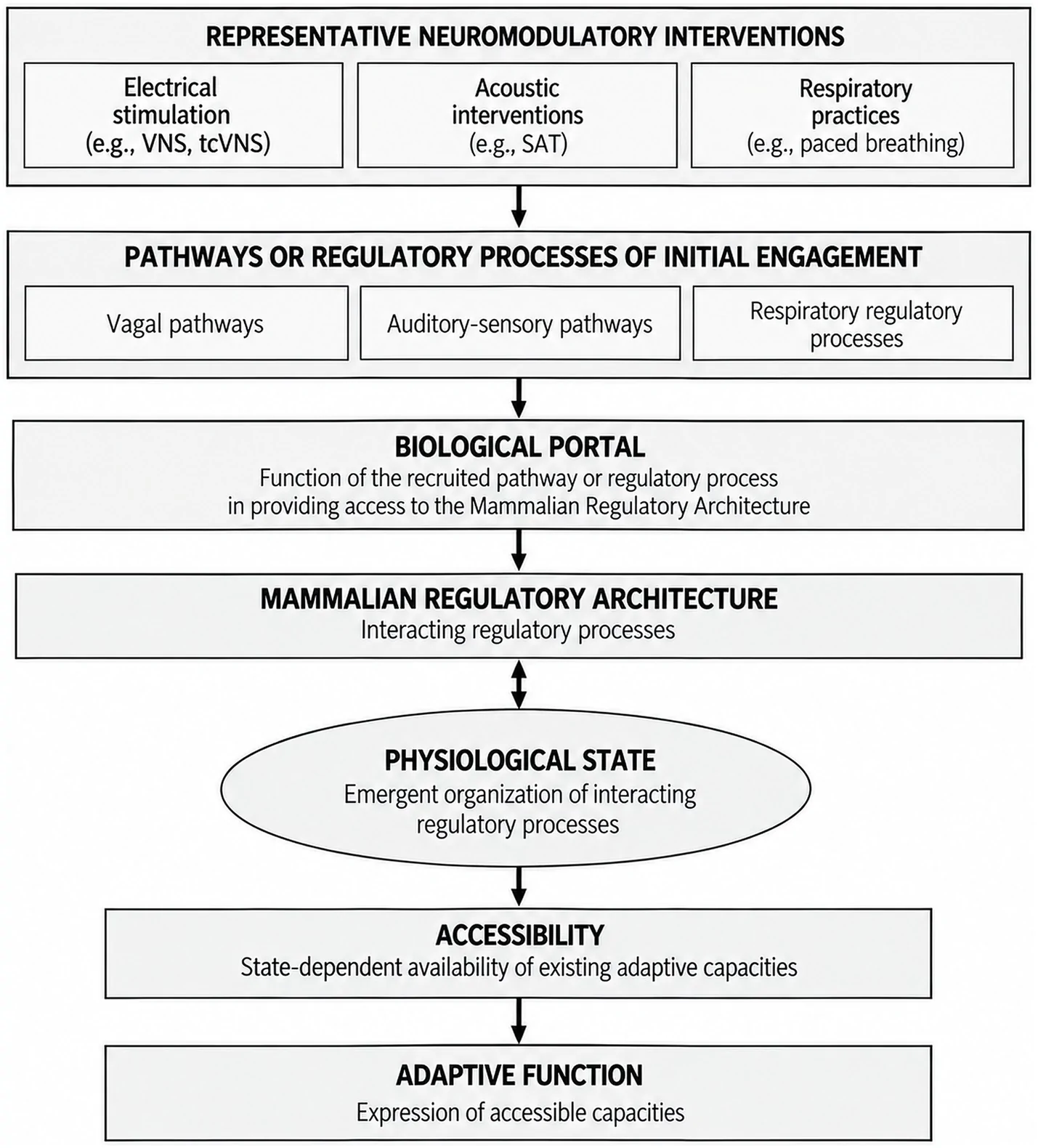
From initial engagement to adaptive function. The model distinguishes initial engagement, biological portal, Mammalian Regulatory Architecture, physiological state, Accessibility, and adaptive function as related but independently testable components. Their relationships should not be inferred from adaptive outcomes alone.

This formulation does not require interventions entering through different biological routes to share pathways or mechanisms, nor does architecture-level convergence require them to become physiologically identical. Initiating pathways, regulatory processes, and mechanisms require independent investigation, and pathway-specific physiological signatures may remain distinguishable. Distinct routes may function as biological portals by influencing regulatory processes participating in the MRA while retaining pathway-specific effects. Pathway-specific effects and architecture-level convergence may therefore coexist and require independent characterization.

Applied to neuromodulation, this distinction generates a direct empirical question: when interventions entering through substantially different biological routes produce overlapping adaptive outcomes, do their effects also converge in aspects of physiological organization? Outcome convergence identifies a phenomenon requiring explanation but does not establish physiological convergence, engagement of identical regulatory processes within the MRA, or a shared mechanism. Testing the architecture-level proposition therefore requires physiological measurement capable of determining whether interventions entering through different routes produce convergent patterns across independently measured physiological processes while preserving the possibility of pathway-specific signatures.

Physiological convergence is not established simply by demonstrating that several physiological variables change following both interventions. Within the MRA, physiological convergence refers to convergence in features of physiological organization. Consistent with physiological state being conceptualized as an emergent property of that organization, relevant evidence concerns the activity of independently measured physiological processes together with their relationships, temporal coordination, and reorganization across changing conditions. Importantly, the temporal patterning of peripheral physiological signals may contain information about the neural processes regulating them.

Earlier psychophysiological research provides methodological and empirical precedents for identifying features of neural regulation expressed in peripheral physiological patterning. Respiratory sinus arrhythmia (RSA), for example, was conceptualized not simply as variability in heart rate but as a peripheral manifestation of neural interactions coordinating respiratory and cardiac control, expressed through vagal regulation of the heart (Porges, 1986). The rhythmic structure of a peripheral physiological signal may therefore provide information about neural regulation as well as the activity of the end organ itself. Physiological time series are also frequently nonstationary, requiring analytical methods that preserve temporally meaningful rhythmic information while accommodating changing baselines (Lewis et al., 2012). Physiological convergence must therefore be sought in dynamic physiological organization rather than inferred from parallel changes in isolated measures.

Coherence and weighted coherence provide approaches to quantifying one dimension of this relational organization (Porges et al., 1980; Porges et al., 1981; Porges and Coles, 1982; Porges, 1986). Applied to simultaneously recorded physiological signals, these methods characterize the extent to which distinguishable processes are coupled within specified frequency domains. Coherence reflects the temporal consistency of the relationship: coupling is stronger when the phase relationship between signals remains stable over time and weaker as that relationship becomes less consistent. It therefore provides information not available from the magnitude of either signal considered independently. Individual differences in respiratory–heart period coherence were associated with different patterns of cardiac reactivity during an attention-demanding task (Porges and Coles, 1982). Importantly, pharmacological blockade with atropine demonstrated that the cholinergic pathways contributing to the respiratory–heart rate pattern expressed as RSA did not similarly determine respiratory–heart period coupling (Porges, 1986). Coupling therefore reflected an organizational feature distinguishable from the cholinergic mechanism contributing to one of its component rhythms. This finding illustrates a principle relevant to the MRA: physiological organization can be empirically characterized without assuming that the relational measure identifies the neural mechanism responsible for that organization.

Experimental perturbation provides an additional strategy for investigating organization. Orthostatic challenge altered cardiovascular coupling differently across frequency bands (Hatch et al., 1986), while frequency-specific oscillatory tilt provided a controlled perturbation through which coupling between an imposed oscillation and the resulting physiological response could be examined (Byrne and Porges, 1992). Individuals with higher baseline RSA, used as an index of cardiac vagal tone, showed a more rapid increase in the targeted low-frequency heart-period rhythm than individuals with lower baseline RSA. Although these findings predate and do not test the MRA, they illustrate an important organizational principle: the physiological organization present at the time of perturbation may influence how the system subsequently responds.

Vagal efficiency (VE) provides a complementary relational measure by quantifying the dynamic functional relationship between RSA and heart period during changing physiological conditions. Rather than treating RSA or heart period as isolated indicators, VE characterizes how changes in cardiac vagal regulation relate to changes in cardiac chronotropy. It therefore illustrates the same methodological principle as coherence: biologically relevant information may reside in relationships among physiological processes rather than solely in the magnitude of individual component measures.

The functional relevance of VE has been demonstrated across physiological and clinical contexts. VE differentiated adolescents with functional abdominal pain disorders and joint hypermobility during postural challenge (Kolacz et al., 2021), was atypically low in children with cyclic vomiting syndrome (Kolacz et al., 2023), and was associated with childhood maltreatment and symptoms of depression and anxiety in college students (Dale et al., 2022). These findings do not establish the MRA; they demonstrate that relationships among physiological processes may reveal differences in regulatory organization not captured by considering component measures independently.

Neither coherence nor vagal efficiency measures the MRA. Nor would any single relational metric provide a universal measure of physiological state. Their relevance is methodological: together, these approaches demonstrate that physiological organization can be investigated through temporal patterning within physiological signals, relationships among distinguishable processes, and changes in those relationships across conditions. Testing the MRA requires extending this relational logic to multivariable physiological organization, including temporal coordination and adaptive reorganization among processes. The relevant measures and relationships should follow the regulatory organization being tested rather than being combined into an assumed global index.

Testing this proposition therefore requires prospective designs capable of characterizing physiological organization across processes and time. A comparison across biological portals could, for example, record respiration, heart period and RSA, and blood pressure continuously during baseline, intervention, a standardized challenge, and recovery, with neuroendocrine or immune indicators sampled at time scales appropriate to those processes. Prespecified analyses could characterize individual signals together with respiratory–cardiac and cardiovascular coupling, temporal coordination, and recovery trajectories across intervention and control conditions. Separate assessments would establish whether particular capacities are present and test their availability under comparable task conditions, while adaptive outcomes would be measured independently. Physiological convergence would be supported by reproducible overlap in prespecified features of physiological organization across distinct routes, potentially alongside route-specific signatures—not by improvement on the same symptom scales or by parallel changes in isolated physiological variables. The selected measures, relationships, and time scales would necessarily follow the regulatory organization being tested and would not constitute a universal score of physiological state.

### 6.1 Convergence across biological portals: an empirical illustration

The Long COVID study (Staats et al., 2026) provides an empirical illustration of outcome convergence across interventions engaging the nervous system through substantially different biological routes. The study was not designed to test the MRA and did not include the multivariable physiological measurements required to evaluate physiological convergence. It therefore does not establish engagement of a common regulatory architecture or a shared mechanism. Rather, the observed outcome convergence identifies a phenomenon that the MRA can organize into testable questions concerning physiological organization, response profiles, and functional significance.

Long COVID is characterized by symptoms spanning multiple functional domains, including fatigue, cognition, sleep, mood, autonomic function, pain, and limitations in daily activity. Symptoms across these domains do not themselves demonstrate disruption of the MRA or identify a common underlying mechanism. Their relevance here is that they provide multiple functional outcomes through which convergence across interventions entering through different biological routes can be evaluated.

In the randomized four-arm study, adults with persistent Long COVID symptoms were assigned to structured acoustic stimulation (SAT), transcutaneous cervical vagus nerve stimulation (tcVNS), their combination, or control. SAT and tcVNS provide markedly different modes of engagement: SAT delivers structured acoustic input through sensory pathways, whereas tcVNS directly stimulates the cervical vagal region and is interpreted primarily in relation to vagal afferent signaling. Within the Portal Model, these substantially different routes are evaluated for their functional significance as biological portals rather than assumed to share an initial pathway or mechanism.

Across the active conditions, within-group analyses showed improvement across multiple symptom and functional domains, whereas the control group showed comparatively little change and anxiety worsened. The pattern was especially notable because SAT and tcVNS entered through substantially different biological routes yet were associated with overlapping multidomain improvement. Differences among the active interventions were not statistically significant. The absence of significant differences among active interventions, however, does not establish their equivalence, nor does similarity in their outcome profiles establish a shared mechanism or physiological organization.

This pattern is therefore most appropriately characterized as outcome convergence. Within the Portal Model, outcome convergence across distinct modes of engagement generates a further question: do these different inputs also produce convergence in aspects of dynamic physiological organization? The study cannot answer that question because the physiological measures required to characterize relationships and temporal coordination among regulatory processes were not collected. The findings therefore identify the phenomenon requiring explanation without establishing the architecture-level process proposed to account for it.

The within-group changes illustrated in Figure 8 should not be interpreted as estimates of randomized treatment effects. The study did not include sham stimulation, and expectancy, attention associated with intervention participation, spontaneous improvement, regression to the mean, and other nonspecific influences cannot be excluded. Figure 8 therefore displays observed within-group outcome patterns rather than attributing those changes to the interventions.

**Figure 8:**
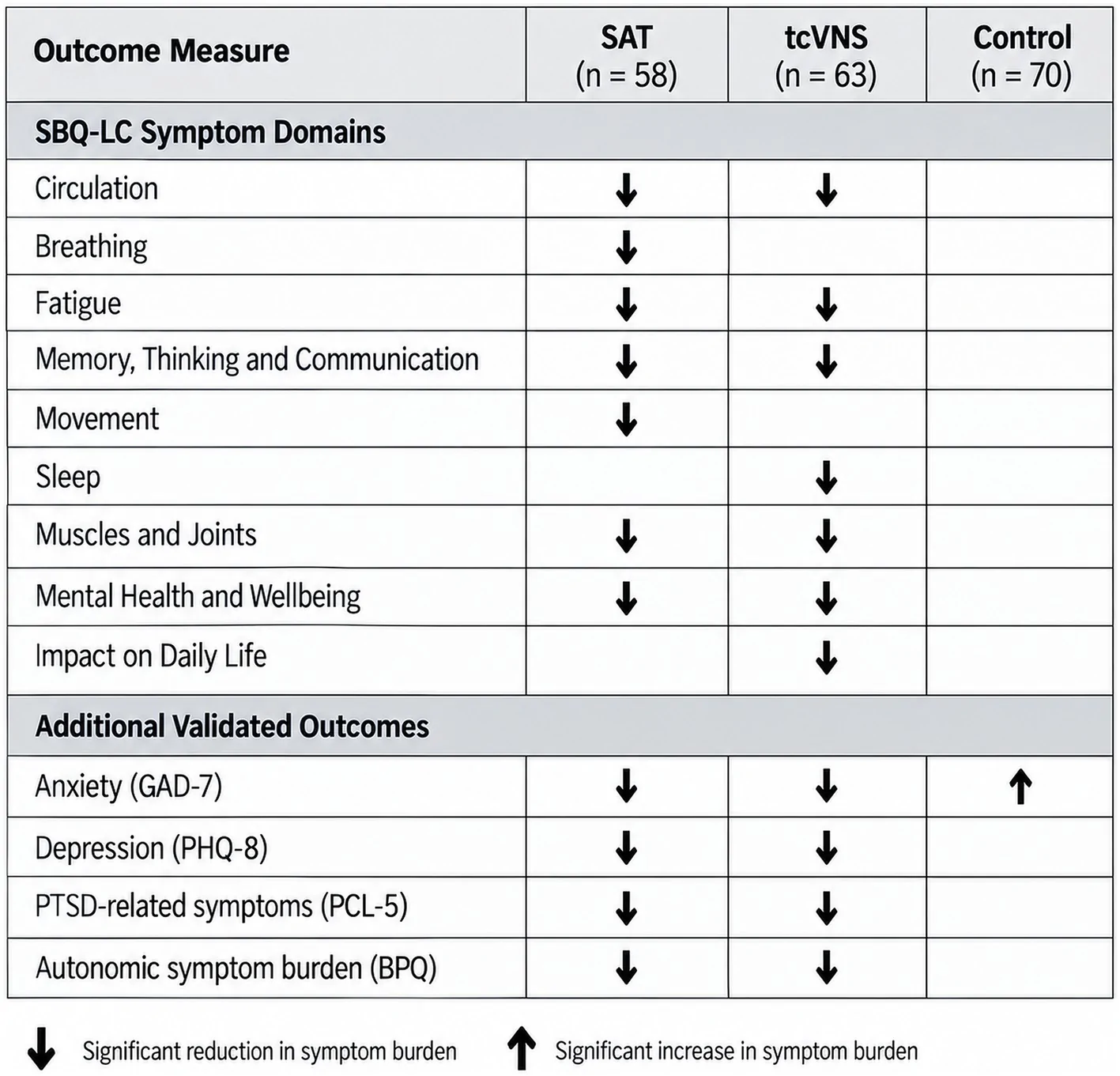
Outcome convergence across distinct biological portals in long COVID. SAT and tcVNS engage distinct biological routes yet show overlapping patterns of significant within-group symptom improvement. Outcome convergence does not establish equivalence, physiological convergence, or a shared mechanism.

The study also did not directly test Accessibility. Improvement in fatigue, cognition, movement, mood, or daily function does not by itself demonstrate that existing capacities became more accessible as physiological state changed. A prospective test would require independent evidence that the relevant capacity exists, characterization of the physiological organization under which its expression is facilitated or constrained, and assessment of whether its availability changes systematically with that organization. The Long COVID outcomes are therefore relevant to the functional domains in which Accessibility may be investigated but do not establish Accessibility itself.

The Combined condition provides an additional opportunity to consider the organizational logic of the Portal Model. In the present study, outcomes in the Combined SAT + tcVNS condition were not significantly different from those in either SAT or tcVNS alone. This finding does not establish equivalence among the active conditions, nor does it identify the physiological organization or mechanisms underlying their outcomes. If portals govern input while the architecture governs the response profile, simultaneous engagement through two distinct portals need not produce a response that is simply additive or greater than either intervention alone. The present study was not designed to determine how the two inputs were integrated within the architecture; this question generates additional empirically distinguishable predictions developed in Section 7.

The Long COVID findings thus transform observed outcome convergence into prospective questions about physiological organization and functional significance. They do not confirm the MRA but identify the next empirical question: whether engagement through distinct biological portals is associated with convergence in physiological organization and whether that organization is systematically related to Accessibility and adaptive function. These questions provide the basis for the empirically distinguishable predictions developed in Section 7.

## 7 Predictions and research directions derived from mammalian Synapomorphy and regulatory architecture

The proposed framework generates four empirically distinguishable predictions concerning physiological convergence across biological portals, physiological state and Accessibility, the functional significance of portal engagement, and evolutionary organization. These predictions derive from relationships proposed within the MRA rather than from any single component of the architecture. Although individual predictions may also be compatible with other regulatory or systems models, the MRA specifies a coordinated set of relationships that can be empirically evaluated. The predictions do not require a rigid unidirectional causal sequence, and each can be evaluated independently. Findings can therefore support, challenge, or refine particular relationships within the framework without requiring acceptance or rejection of the architecture as a whole.

A first prediction concerns physiological convergence across biological portals. When interventions entering through substantially different biological routes produce convergent adaptive outcomes that are hypothesized to reflect their engagement of the MRA, they should also produce convergence in aspects of physiological organization despite differences in their initiating pathways and mechanisms. The prediction is not that different interventions become physiologically identical. Rather, convergence should be detectable in features of physiological organization across independently measured processes, including their relationships and temporal coordination, while route-specific physiological signatures may remain distinguishable. Thus, convergence at the level of broader physiological organization can coexist with specificity at the level of the initiating pathway or mechanism.

Physiological convergence therefore provides a more discriminating test of architecture-level convergence than similarity of adaptive outcomes alone. Repeated findings of convergent adaptive outcomes without convergence in prespecified features of physiological organization would challenge the proposition that those outcomes reflect convergence at the architecture level. Such findings would not negate the observed outcome convergence or pathway-specific mechanisms; they would constrain the architecture-level explanation proposed for their similarity.

The prediction also extends to simultaneous engagement through more than one biological portal. Because portals govern input while the architecture governs the response profile, the physiological organization associated with combined portal engagement cannot be assumed to represent the simple summation of responses associated with each portal independently. It may resemble the organization associated with one portal, share features associated with both, or form a distinguishable organizational response, depending on how the inputs are integrated within the architecture. Comparing single- and combined-portal engagement therefore provides an experimental strategy for determining how distinct inputs are integrated within the proposed architecture.

A second prediction concerns the relationship between physiological state and Accessibility. If Accessibility is state dependent, changes in physiological organization should be systematically related to changes in the availability and expression of independently established capacities. The prediction concerns relationships among independently characterized variables rather than an inference of physiological state from adaptive outcomes or an inference of Accessibility from changes in performance alone. Evidence relevant to this prediction therefore requires physiological measures capable of characterizing changes in physiological organization, including, where relevant, relationships and temporal coordination among physiological processes, together with measures that establish the presence of relevant capacities and assess their availability for expression under comparable conditions.

Repeated-measures and longitudinal designs can evaluate whether changes in physiological organization precede, covary with, or follow changes in the availability and expression of established capacities. The framework does not require a strictly unidirectional sequence because physiological state emerges from reciprocal relationships among regulatory processes, and adaptive behavior may itself alter the conditions within which physiological regulation occurs. Temporal precedence and covariance therefore provide different information and should not be treated as equivalent evidence for direction of influence. The proposed relationship would be supported by systematic associations between independently characterized physiological organization and independently assessed changes in the availability of established capacities, and would require reconsideration if those relationships were not observed.

A third prediction concerns the functional significance required to identify engagement through a biological route as portal engagement. Whereas physiological convergence compares the consequences of engagement through different biological routes, this prediction asks whether engagement through a particular route conceptualized as a biological portal produces changes in physiological organization that are systematically related to Accessibility and adaptive function. A pathway does not acquire functional significance as a portal merely because its engagement is followed by an adaptive outcome. Rather, its proposed portal significance requires evidence linking engagement through that route to changes in physiological organization and linking those changes to Accessibility or adaptive function.

If engagement through a route conceptualized as a biological portal influences regulatory processes participating in the MRA, that engagement should be associated with predicted changes in physiological organization. The functional significance of those changes can then be evaluated through their systematic relationships with Accessibility and adaptive function. This provides an empirical bridge from an identifiable pathway of initial engagement to its proposed functional significance within the broader regulatory architecture. Repeated adaptive improvement in the absence of the predicted changes in physiological organization would weaken the proposed architecture-level explanation for that intervention. Such a result would not demonstrate that the intervention is ineffective or that its initiating pathway is incorrectly identified. It would instead indicate that the proposed relationship among portal engagement, physiological organization, Accessibility, and adaptive function requires modification or an alternative explanation.

A fourth prediction concerns the evolutionary organization hypothesized from identified mammalian synapomorphies and comparative evidence. The framework does not predict that individual autonomic, communicative, caregiving, or social processes are unique to mammals. Rather, it predicts that mammalian evolution was associated with a distinctive functional integration of conserved regulatory processes with mammalian specializations and with regulatory demands associated with nursing, prolonged developmental dependency, caregiving, co-regulation, and reciprocal social communication. The evolutionary proposition therefore concerns a hypothesized reorganization of relationships among biological processes rather than the evolutionary origin of every component participating in that organization.

Comparative evaluation should consequently focus on the proposed pattern of functional organization associated with mammalian evolution rather than on the presence or absence of isolated structures, pathways, or functions across vertebrate species. Homologous antecedents in non-mammalian vertebrates are compatible with the framework because the presence of an evolutionarily conserved component does not establish that its functional relationships remained unchanged. Conversely, analogous functions across species do not establish identical regulatory mechanisms. Identified mammalian synapomorphies provide evolutionary anchors from which changes in regulatory relationships can be investigated, with multiple synapomorphies providing complementary points of comparison for evaluating whether the regulatory demands accompanying mammalian evolution were associated with changes in integrated regulatory organization.

Identifying a mammalian synapomorphy does not itself establish the regulatory organization proposed by the MRA. The empirical question is whether comparative evidence supports a pattern of regulatory relationships associated with mammalian evolution that is consistent with the proposed architecture. Evidence that the proposed pattern of functional integration is not distinctively associated with mammalian organization would challenge the evolutionary proposition even if the individual structures or functions incorporated into the model were correctly described.

The four predictions evaluate complementary but empirically separable relationships within the proposed architecture. Evidence supporting one relationship does not establish the others. Physiological convergence across different routes could occur without establishing the proposed relationship between physiological state and Accessibility, while state-dependent changes in Accessibility could be demonstrated without convergence across biological portals. Similarly, engagement through a particular route could influence broader physiological organization without establishing the evolutionary interpretation proposed for the MRA. Comparative evidence may, in turn, refine the evolutionary boundaries of the architecture without altering experimentally demonstrated regulatory relationships. The framework therefore links these propositions while preserving their independence as empirical questions.

Taken together, these predictions make the MRA experimentally testable across distinct levels of biological organization. The framework is evaluated not by whether any single pathway, physiological measure, or adaptive outcome confirms it, but by whether the predicted relationships among portal engagement, physiological organization, Accessibility, adaptive function, and evolutionary organization are empirically observed. Neuromodulation thereby becomes not only a means of influencing function but an experimental strategy for testing how regulatory organization changes when identifiable biological routes are engaged.

## 8 Conclusion

The Mammalian Regulatory Architecture expands the scientific and therapeutic scope of neuromodulation by extending inquiry beyond the pathway initially engaged to the regulatory organization in which that pathway participates. Neuromodulation appropriately focuses on anatomically and physiologically identifiable targets, pathways, and mechanisms, and the MRA retains that biological specificity. However, identifying the pathway through which an intervention enters the nervous system does not, by itself, specify the broader functional response that follows. The pathway specifies the route of initial engagement. Its significance as a portal concerns how engagement through that route influences physiological organization within the architecture. The resulting response profile reflects the broader pattern of physiological and functional change associated with that engagement. The central shift is therefore from treating the initially engaged pathway as sufficient to explain the response to investigating how pathway-specific engagement influences physiological organization. Physiological state emerges from this organization and influences the conditions under which existing capacities become accessible for recruitment and expression. The functional consequences of neuromodulation therefore cannot be inferred from the pathway of initial engagement alone.

This shift changes what is required scientifically. Measuring more physiological variables is not sufficient if they continue to be analyzed and interpreted as independent components. Testing architecture-level hypotheses requires dynamic, multivariable approaches capable of characterizing how interacting regulatory processes coordinate and reorganize in response to intervention and changing demands. The relevant information may reside not only in the magnitude of individual responses but also in their relationships and temporal organization across intervention, challenge, and recovery. Neuromodulation is particularly informative in this context because it permits an identifiable biological route to be experimentally engaged while the resulting reorganization of broader physiological processes is characterized. The intervention can therefore function as a controlled perturbation of the regulatory architecture: the route of engagement is specified, while the resulting response profile provides an opportunity to investigate how that input is integrated within the architecture, whether physiological organization changes, and how those changes are related to Accessibility and adaptive function.

The same framework changes how intervention can be conceptualized. Compromised function may reflect a capacity that is absent, impaired, or insufficiently developed; an existing capacity whose Accessibility is constrained under prevailing physiological conditions; or both. This distinction changes the therapeutic question. When a capacity is deficient, interventions based on learning, training, or neural exercise may appropriately focus on developing, strengthening, or restoring that capacity. When the capacity is present but its Accessibility is constrained, neuromodulation may be directed toward altering the physiological organization limiting its recruitment and expression. Neuromodulation can therefore be investigated as a biological portal for altering physiological organization in ways that change Accessibility to existing capacities. By changing the physiological conditions under which capacities become available for recruitment and expression, neuromodulation may influence not only symptoms and compromised function but also Accessibility to capacities involved in learning, decision-making, attention, communication, and other forms of adaptive behavior.

These classes of intervention need not be mutually exclusive. Their potential complementarity is illustrated by evidence that transcutaneous cervical vagus nerve stimulation delivered during second-language training accelerated vocabulary acquisition, with benefits maintained at a subsequent test without stimulation (Miyatsu et al., 2024). In this example, the information and experience required for learning were provided through training, while neuromodulation was associated with enhanced acquisition. From the perspective of the MRA, such findings raise the testable hypothesis that altering physiological organization and changing Accessibility may create conditions that facilitate engagement with interventions based on learning, training, or neural exercise and thereby influence their effectiveness. This perspective expands intervention design by making the physiological organization present before and during intervention relevant to how interventions are selected, delivered, sequenced, or combined.

Several takeaways follow from this architecture-centered formulation of neuromodulation.

1. The pathway of initial engagement does not specify the broader functional response. The pathway identifies the biological route through which an intervention initially engages the nervous system, while its significance as a portal concerns how engagement through that route influences processes participating in the architecture. The route of engagement and the resulting response profile can therefore be characterized independently rather than inferred from one another.
2. Physiological state is an emergent property of physiological organization. Physiological state therefore cannot be established by a single physiological variable. Physiological organization can be empirically characterized through multivariable measurement of interacting physiological regulatory processes, including their relationships, temporal coordination, and reorganization across intervention, challenge, and recovery.
3. The presence of a capacity does not establish its Accessibility. An existing capacity may remain present while its availability for recruitment and expression changes with physiological state. Accessibility can therefore be evaluated by independently establishing that a capacity is present and determining whether its availability for expression changes systematically in relation to independently characterized physiological organization.
4. Neuromodulation can function as a controlled perturbation of the regulatory architecture. Engagement through an identifiable biological route provides an experimental means of determining whether physiological organization changes and whether those changes are systematically related to Accessibility and adaptive function. The route of engagement, physiological organization, Accessibility, and adaptive function can therefore be characterized independently rather than inferred from one another.
5. Intervention design can be investigated in relation to physiological organization rather than symptoms or diagnosis alone. The portal engaged, dose, duration, timing, sequencing, combination with other interventions, and the physiological organization present at engagement become experimentally relevant variables. Physiological organization may also influence Accessibility to capacities required for engagement with learning, training, or neural exercise, making the effectiveness of these interventions itself a testable function of physiological conditions. These relationships can be evaluated across interventions, physiological conditions, and single- versus combined-portal engagement.

Together, these principles expand what neuromodulation can seek to understand and accomplish. The advance is not simply to identify more targets, engage more pathways, or measure more variables, but to determine how biologically specific pathways participate within an integrated regulatory architecture and how that organization supports the Accessibility and expression of adaptive capacities. Knowledge gained through this approach can inform more precise strategies for selecting, timing, sequencing, and combining interventions in relation to the physiological organization present at the time of engagement. Neuromodulation can thereby become both an intervention and an experimental probe: a means of influencing physiological organization and of investigating how integrated regulatory organization supports physiological state, Accessibility, and adaptive function.
